# A two-step lineage reprogramming strategy to generate functionally competent human hepatocytes from fibroblasts

**DOI:** 10.1038/s41422-019-0196-x

**Published:** 2019-07-03

**Authors:** Bingqing Xie, Da Sun, Yuanyuan Du, Jun Jia, Shicheng Sun, Jun Xu, Yifang Liu, Chengang Xiang, Sitong Chen, Huangfan Xie, Qiming Wang, Guangya Li, Xuehui LYU, Hui Shen, Shiyu Li, Min Wu, Xiaonan Zhang, Yue Pu, Kuanhui Xiang, Weifeng Lai, Peng Du, Zhenghong Yuan, Cheng Li, Yan Shi, Shichun Lu, Hongkui Deng

**Affiliations:** 1 School of Basic Medical Sciences, State Key Laboratory of Natural and Biomimetic Drugs, Peking University Health Science Center and the MOE Key Laboratory of Cell Proliferation and Differentiation, College of Life Sciences, Peking-Tsinghua Center for Life Sciences, Peking University, Beijing, 100191 China; 2 State Key Laboratory of Chemical Oncogenomics, School of Chemical Biology & Biotechnology, Peking University Shenzhen Graduate School, Shenzhen, Guangdong 518055 China; 3 Center for Bioinformatics, Peking University, Beijing, 100871 China; 4 MOE Key Laboratory of Cell Proliferation and Differentiation, College of Life Sciences, Peking-Tsinghua Center for Life Sciences, Peking University, Beijing, 100871 China; 5 Shanghai Public Health Clinical Center, Shanghai, 201508 China; 6 Hangzhou Repugene Technology Co,. Ltd, Hangzhou, China; 7 School of Basic Medical Sciences, Peking University Health Science Center, Beijing, 100191 China; 8 Key Laboratory of Medical Molecular Virology, Fudan University, Shanghai, 200032 China; 9 Department of Hepatobiliary Surgery, Chinese PLA General Hospital, Beijing, 100853 China

**Keywords:** Stem cells, Transdifferentiation

## Abstract

Terminally differentiated cells can be generated by lineage reprogramming, which is, however, hindered by incomplete conversion with residual initial cell identity and partial functionality. Here, we demonstrate a new reprogramming strategy by mimicking the natural regeneration route, which permits generating expandable hepatic progenitor cells and functionally competent human hepatocytes. Fibroblasts were first induced into human hepatic progenitor-like cells (hHPLCs), which could robustly expand in vitro and efficiently engraft in vivo. Moreover, hHPLCs could be efficiently induced into mature human hepatocytes (hiHeps) in vitro, whose molecular identity highly resembles primary human hepatocytes (PHHs). Most importantly, hiHeps could be generated in large quantity and were functionally competent to replace PHHs for drug-metabolism estimation, toxicity prediction and hepatitis B virus infection modeling. Our results highlight the advantages of the progenitor stage for successful lineage reprogramming. This strategy is promising for generating other mature human cell types by lineage reprogramming.

## Introduction

The generation of desired functional cells is a long-standing goal of stem cell research and regenerative medicine. In the past few decades, extensive efforts have been made to develop various strategies to produce functional cells by differentiation from pluripotent stem cells or conversion from a distinct cell type by lineage reprogramming.^1,2^ However, for many functional lineages, these strategies encountered a major challenge: the cells generated in vitro rarely obtained mature cell identity and functionality.^3,4^ Specifically, most of these cells retained the expression of fetal markers and/or were lacking in the expression of adult markers, consequently leading to an immature cell state.^5,6^ Therefore, it remains a challenge to develop a new approach to generate desired cells in vitro that resemble their counterparts isolated from the native tissue.

Direct lineage reprogramming can induce cell fate conversion between differentiated cell types across developmental lineages. Different from pluripotent stem cell differentiation, direct lineage reprogramming demands relatively fewer clues from human embryonic development, especially late fetal stages that remain largely unknown. Previous studies showed the feasibility of direct lineage reprogramming to generate various human cell types in a straightforward manner in vitro.^1,7,8^ However, this direct strategy suffers from several shortcomings, including the residual memory of the initial cells and limited function of target cells.^9^ Zaret and colleagues had shown the reprogramming barriers, including packed H3K9me3 heterochromatin domains, as the main reasons for inadequate silencing of initial cell identity genes and impaired activation of gene regulatory networks of the target cell type.^10^ A new strategy to overcome these barriers would greatly improve the functionality of cells generated by lineage reprogramming.

An important clue to overcome reprogramming barriers is provided during tissue regeneration. In the wound of lower animals, the remaining differentiated cells can repair the injured tissue by first de-differentiation to proliferative progenitors, which is followed by extensive expansion, and then they re-differentiate to functionally mature cells that reform the tissue.^11,12^ Notably, the proliferation of progenitors that possess a plastic gene expression profile is associated with global epigenetic remodeling, which permits these progenitors to respond to differentiation signals for re-differentiation to the functionally mature state.^13–15^ This suggests two important principles: that progenitor cells with relatively open chromatin architectures are more amenable to cell-fate induction, and that proliferation of such cells allows the generation of abundant functional cells. These in-vivo regeneration principles could be used for developing a new lineage reprogramming approach in vitro.

**Fig. 1:**
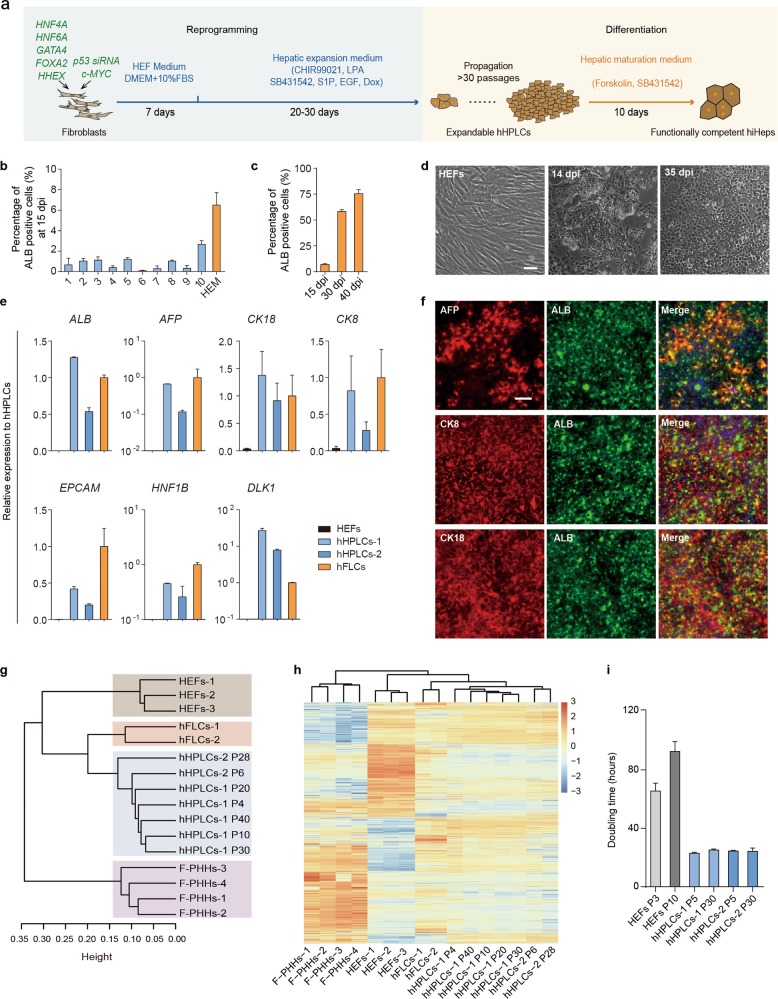
Generation of human hepatic progenitor-like cells (hHPLCs) from fibroblasts by defined factors. **a** Scheme of the two-step reprogramming process. **b** Quantification of reprogrammed ALB^+^ cells in different hepatic progenitor culture media at 15 dpi. *n* = 2. **c** Quantification of reprogrammed ALB^+^ cells in optimized HEM at different time points. *n* = 3. **d** Bright field images of initiating HEFs, reprogrammed epithelial colonies at 14 dpi and epithelial cells at 35 dpi. **e** RT-qPCR analysis of hHPLCs, HEFs and hFLCs for human hepatic progenitor markers. Relative expression was normalized to hFLCs. *n* = 2. **f** Co-Immunofluorescence staining of hHPLCs for human hepatic progenitor markers AFP, CK8 and CK18 with ALB. **g** Hierarchical clustering of global gene expression of HEFs, hFLCs, F-PHHs and hHPLCs at different passages. **h** Heatmap of differentially expressed genes among HEFs, hFLCs and hHPLCs at different passages. **i** Population doubling time of hHPLCs at P5 and P30 as well as of HEFs at P3 and P10. *n* = 3. For all measurements, ‘*n*’ represents the number of biological replicates. The scale bars represent 50 μm. Data are presented as mean ± SEM

In this study, we developed a new two-step strategy mimicking a natural cell-fate conversion route during regeneration to generate expandable hepatic progenitor cells and functionally competent human hepatocytes (Fig. 1a). In the first step, we used a combination of 5 hepatic transcription factors to reprogram fibroblasts into proliferative hepatic progenitor cells, which could then be selected and expanded in a newly developed hepatic expansion medium. Next, hepatic progenitor cells were chemically induced into functionally competent hepatocytes. Comprehensive analysis revealed that hepatocytes generated by this two-step strategy highly resembled freshly isolated primary hepatocytes in cell identity and functionality, with broad application potential for drug discovery and liver disease modeling.

## Results

### Generation of proliferative human hepatic progenitor cells

To generate human hepatic progenitor cells from human embryonic fibroblasts (HEFs), we selected a pool of transcription factors (TFs) as candidates based on (1) their importance in hepatic organogenesis, and (2) computational analysis of the highly expressed TFs in human fetal liver cells (hFLCs), a specified type of human hepatic progenitor cells that gives rise to hepatocytes in adult liver (Supplementary information, Table S1). Among these transcription factors, four TFs (4-TFs) were selected as the base combination, which included GATA4 and FOXA2 for their function as pioneering factors to unveil heterochromatin during endodermal patterning,^15^ as well as HNF4A and HNF6A that were selected as hepatic fate determinant factors.^8^ In addition, to overcome proliferation arrest and cell death during reprogramming, expression of *c-MYC* and *P53* small interfering RNAs (*P53* siRNAs) was coupled with 4-TFs, a strategy that was employed in our previous reprogramming studies.^8^ Following overexpression of 4-TFs in HEFs, reverse transcription quantitative polymerase chain reaction (RT-qPCR) showed up-regulation of *ALB* and *AFP* in the reprogrammed cells (Supplementary information, Fig. S1a). We further screened various additional transcription factors based on 4-TFs. Analysis of the expression of key transcription factors in hepatic progenitors (*HNF1B* and *LRH1*) and an epithelial marker (*CDH1*) showed that *HHEX* outperformed all the other TFs at the early stage of reprogramming (Supplementary information, Fig. S1b). Furthermore, the combination of 4-TFs and HHEX (termed as 5-TFs) boosted the generation of ALB^+^ AFP^+^ double-positive cells, which was observed within 10 days in the fibroblast culture (Supplementary information, Fig. S1c). These data suggest that *HHEX* effectively promoted the hepatic lineage reprogramming.

To select and expand these ALB^+^AFP^+^ cells, we tested 10 different media (M1 to M10, see more details in Supplementary information, Table S2) that have been reported to expand hepatic progenitor cells. Among these media, M10, a medium used to expand mouse hepatic progenitor cells, gave the highest yield of 2.7% (±0.3%) ALB^+^ cells at 15 days post-infection (dpi) (Fig. 1b). Based on M10, we further improved the hepatic progenitor expansion condition by substituting basal medium and supplements, and optimizing the small molecule combinations, and finally obtained a hepatic expansion medium (HEM). In this new HEM, epithelial colonies were efficiently generated and ALB^+^ cells were robustly expanded, accounting for ~75% of all cells at 40 dpi (Fig. 1c, d; Supplementary information, Fig. S1d). During this reprogramming process, the expression of fibroblast markers *COL1A1* and *THY1* was downregulated in 5-TFs-overexpressing HEFs. Meanwhile, the expression of hepatic progenitor markers, including *ALB*, *AFP* and *EpCAM*, were greatly upregulated (Supplementary information, Fig. S1e). Additionally, we found that all 5 transcription factors were indispensable for the generation of hepatic progenitors (Supplementary information, Fig. S1f). Collectively, these data indicate that we have established an efficient system to convert human fibroblasts to the hepatic cells with proliferative progenitor features, termed as human hepatic progenitor-like cells (hHPLCs).

We further confirmed the expression of a panel of hepatic progenitor markers by RT-qPCR in hHPLCs including *ALB*, *AFP*, *CK8*, *CK18*, *EPCAM*, *HNF1B* and *DLK1* (Fig. 1e). The co-expression of ALB with AFP, CK8 and CK18 was further validated by immunofluorescence staining (Fig. 1f). Global gene expression analysis by RNA sequencing showed that hHPLCs shared a similar gene expression pattern to that of hFLCs, but was distinct from the initiating HEFs and freshly isolated primary human hepatocytes (F-PHHs) (Fig. 1g, h). In addition, genes that are known to be enriched in hepatic progenitor cells were greatly upregulated in hHPLCs (Supplementary information, Fig. S1g). Collectively, these results indicate that hHPLCs obtained the hepatic progenitor identity.

### hHPLCs efficiently engraft and expand in mouse liver

**Fig. 2:**
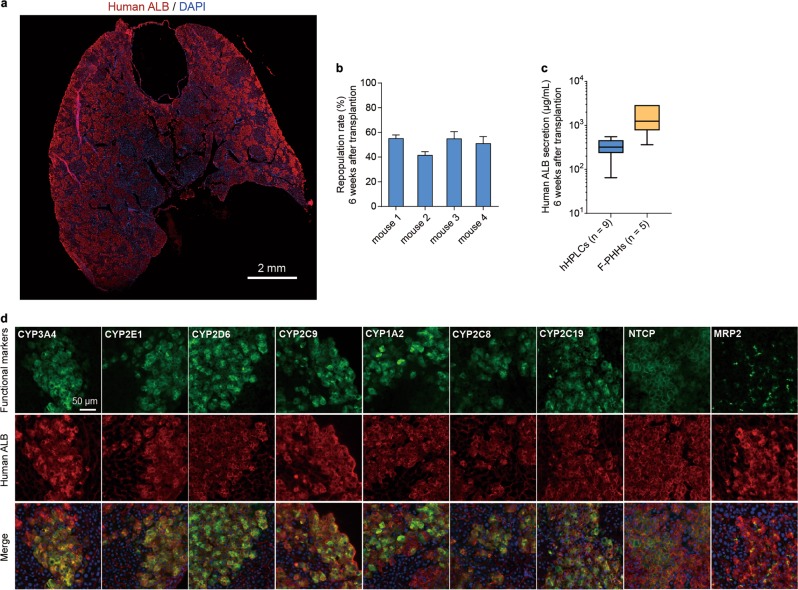
Maturation of hHPLCs in vivo. **a** Spliced immunofluorescence staining of human ALB in hHPLC-engrafted URG mouse liver. The scale bars represent 2 mm. **b** The repopulation rate of hHPLCs in four mice was determined by ALB immunostaining 6 weeks after transplantation. **c** Human ALB secretion at 6 weeks post-transplantation from mice transplanted with hHPLCs (*n* = 9) and F-PHHs (*n* = 5). **d** Immunofluorescence staining of human ALB co-expression with human functional hepatic genes (CYP3A4, CYP2E1, CYP2D6, CYP2C9, CYP1A2, CYP2C8, CYP2C19, NTCP and MRP2) in hHPLC-engrafted URG mouse liver. For all measurements, ‘*n*’ represents the number of biological replicates. The scale bars represent 50 μm. Data are presented as mean ± SEM

To identify whether hHPLCs possessed the capacity to engraft and expand in vivo, we transplanted them into the Tet-uPA (urokinase-type plasminogen activator)/Rag2^−/−^/γc^−/−^ liver injury mouse model.^8,16^ At 6-week after transplantation, we first analyzed the expression of human ALB in the recipient mouse liver by immunofluorescence staining (Fig. 2a). The results showed a robust engraftment of hHPLCs in mouse liver, indicated by an ~50% presence of cells expressing human ALB (Fig. 2a, b). This efficient repopulation rate of mouse liver by hHPLCs was consistent with the secreted human ALB levels in mouse serum (Fig. 2c). Importantly, in mouse liver transplanted with hHPLCs, human ALB^+^ cells expressed mature hepatocyte markers, including a series of CYP450 enzymes that are known to metabolize more than 80% of marketed drugs^17^ (Fig. 2d). In addition, more than 50% of human ALB^+^ cells expressed hepatobiliary transporter MRP2, indicating that hHPLC-derived hepatocytes were polarized in vivo (Fig. 2d). We also observed the robust expression of HBV receptor NTCP in human ALB^+^ cells, which resulted in the formation of multiple NTCP^+^ALB^+^ human hepatic islands in mouse liver (Fig. 2d). We next analyzed the tumorigenicity of hHPLCs by subcutaneously transplanting hHPLCs into the immunocompromised NOD-Prkdc^scid^ Il2rg^null^ (NPG) mice. Mice that transplanted with hHPLCs did not develop tumors up to 12 weeks, however all of the HepG2-transplated mice developed tumors 2–3 weeks post-transplantation (Supplementary information, Fig. S1i, j). Collectively, these data show that hHPLCs possess robust capacity to efficiently engraft and expand in vivo with functionally mature features, permitting the generation of humanized liver mouse models in large-scale.

### hHPLC-derived hiHeps resembled freshly isolated primary hepatocytes in cell identity and functionality

**Fig. 3:**
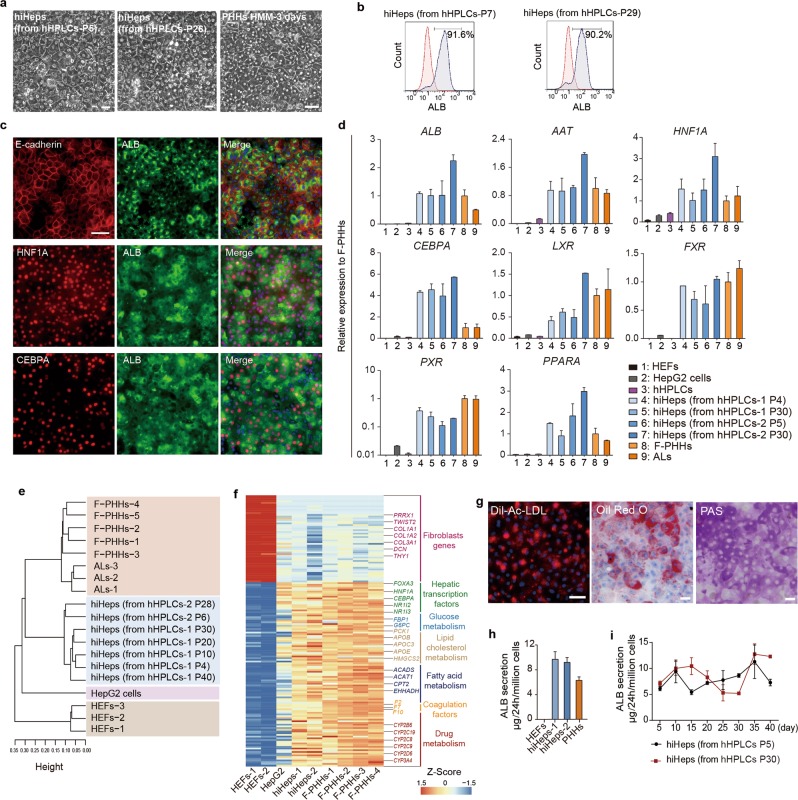
hiHeps recapitulate mature hepatocyte phenotypes as PHHs in vitro. **a** Bright field images show the polygonal morphology of hiHeps derived from hHPLCs-P5 and hHPLCs-P26 and freshly isolated primary hepatocytes cultured in HMM for 3 days. **b** Flow cytometry analysis of ALB positive cells in hiHeps derived from hHPLCs-P7 and hHPLCs-P29. **c** Co-immunofluorescence staining of E-cadherin, HNF1A and CEBPA with ALB in hiHeps derived from hHPLCs-P5. **d** RT-qPCR analysis of major mature hepatocyte functional genes in HEFs (*n* = 3), HepG2 cells (*n* = 2), hHPLCs (*n* = 2), hiHeps derived from hHPLCs at different passages (*n* = 2), F-PHHs (*n* = 5) and ALs (*n* = 4). Relative expression was normalized to F-PHHs. **e** Hierarchical clustering of global gene expression of HepG2 cells, HEFs, F-PHHs, ALs and hiHeps derived from hHPLCs at different passages. **f** Heatmaps of fibroblast genes, hepatic transcription factors and functional hepatocyte genes involved in glucose metabolism, lipid cholesterol metabolism, fatty acid metabolism, coagulation and drug metabolism in HEFs, hiHeps and F-PHHs. **g** Essential hepatic functions in hiHeps: LDL uptake (left), Oil Red O staining (middle) and PAS staining (right). **h** ALB secretion in HEFs, hiHeps and PHHs analyzed by ELISA. *n* = 3. **i** Dynamic monitoring of ALB secretion in hiHeps and PHHs analyzed by ELISA. *n* = 3. For all measurements, ‘*n*’ represents the number of biological replicates. The scale bars represent 50 μm. Data are presented as mean ± SEM

We next investigated the capability of hHPLCs to generate mature hepatocytes in vitro. Most recently, we demonstrated a five-chemical (5C) condition to maintain the long term in vitro function of primary hepatocytes.^18^ In light of this study, we developed a hepatocyte maturation medium (HMM) by adopting key components of the 5C condition, the combination of a cAMP activator and a TGFβ inhibitor, which played the essential role in capturing and stabilizing mature functions of primary human hepatocytes (Supplementary information, Fig. S2a–c). After cultured in HMM for 10 days, hHPLCs formed typical polygonal morphology similar to cultured primary hepatocytes, and the ALB^+^ cells accounted for over 90% of cells in culture as detected by flow cytometry analysis (Fig. 3a, b). In addition, the ALB^+^ cells co-expressed E-cadherin and transcription factors HNF1A and CEBPA (Fig. 3c; Supplementary information, Fig. S2d). Furthermore, hHPLCs cultured in HMM for 10 days showed a dramatic upregulation of major mature hepatocyte genes, including *ALB*, *AAT*, and key hepatic transcription factors, at levels that were comparable to that of F-PHHs and adult liver tissues (ALs) (Fig. 3d). Therefore, we termed the cells differentiated from hHPLCs in HMM as human induced hepatocytes (hiHeps).

We next examined whether the global gene expression pattern of hiHeps resembled that of freshly isolated primary human hepatocytes. Hierarchical clustering revealed that hiHeps clustered closely with F-PHHs and ALs, but were distinct from HepG2 cells and HEFs (Fig. 3e). Importantly, the expression of key hepatic TFs was upregulated, and the expression of fibroblast marker genes was undetectable in hiHeps (Fig. 3f). Moreover, the expression levels of genes involved in glucose metabolism, lipid cholesterol metabolism, fatty acid metabolism and drug metabolism were similar between hiHeps and F-PHHs (Fig. 3f). Overall, the activation of hepatocyte gene regulatory networks revealed the establishment of the hepatocyte-identity in hiHeps.

Function characterization of hiHeps showed that they were competent for low-density lipoprotein (LDL) uptake (Fig. 3g). The accumulation of fat droplets and glycogen synthesis in hiHeps were observed by Oil Red O and Periodic Acid-Schiff (PAS) staining, respectively (Fig. 3g), and the albumin secretion level in hiHeps was similar to that of PHHs (Fig. 3h). Additionally, we found that HMM supported not only maturation, but also the functional maintenance of hiHeps. RT-qPCR analysis showed that a panel of mature hepatocyte markers was upregulated and further maintained for at least one month in HMM (Supplementary information, Fig. S2e). Meanwhile, the progenitor marker AFP was dramatically downregulated with prolonged culture in HMM (Supplementary information, Fig. S2e). Furthermore, the ALB secretion from hiHeps was also maintained for at least one month (Fig. 3i).

### Comparable drug metabolism ability between hiHeps and primary human hepatocytes

**Fig. 4:**
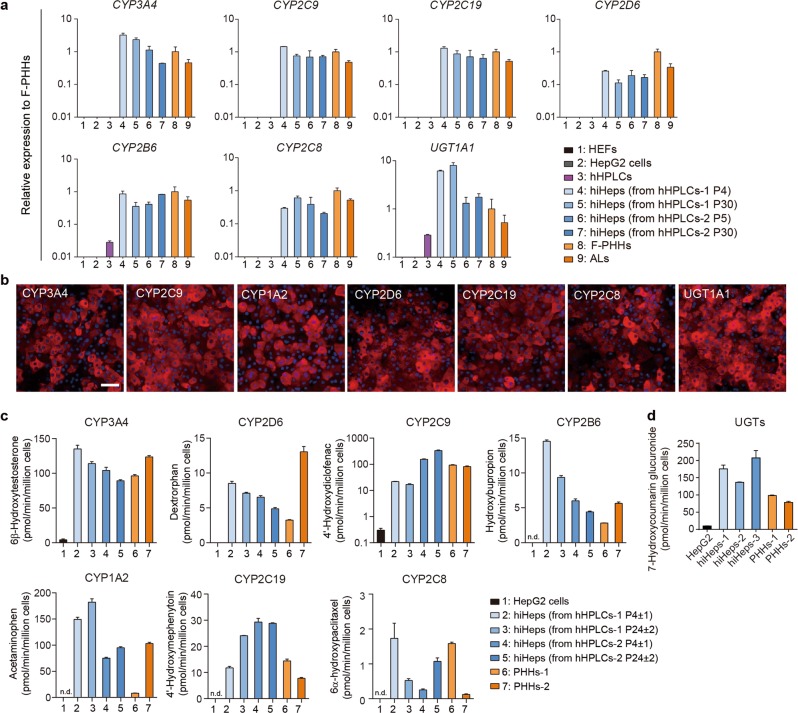
Comparable CYP450 drug-metabolizing activities of hiHeps to PHHs. **a** RT-qPCR analysis of major CYP450s and UGT1A1 in HEFs (*n* = 3), HepG2 cells (*n* = 2), hHPLCs (*n* = 2), hiHeps derived from hHPLCs at different passages (*n* = 2), F-PHHs (*n* = 5) and ALs (*n* = 4). Relative expression was normalized to F-PHHs. **b** Immunofluorescence staining of key CYP450 enzymes (CYP3A4, CYP2C9, CYP2C19, CYP2C8, CYP2D6, CYP1A2) and UGT1A1 in hiHeps derived from hHPLCs at P5. **c** UPLC/MS/MS analysis in hiHeps derived from hHPLCs at different passages, HepG2 cells and F-PHHs for the drug-metabolic activities of 7 CYP450s. *n* = 3. **d** UPLC/MS/MS analysis in hiHeps, HEFs cells and F-PHHs for the drug-metabolic activities of UGTs. *n* = 3. Data not detected were labeled as “n.d.”. For all measurements, ‘*n*’ represents the number of biological replicates. The scale bars represent 50 μm. Data are presented as mean ± SEM

Drug-metabolizing capacity is one of the most important features of mature human hepatocytes; however most in vitro-generated hepatocytes are deficient in this ability.^7,8,19–21^ We sought to examine the drug-metabolizing ability of hiHeps in HMM. First, the gene expression level of key participants involved in the network of drug metabolism was found to be comparable between hiHeps and F-PHHs, which included phase I cytochrome P450 enzymes (CYP450s), phase II UDP-glucuronosyltransferases (UGTs), phase III transporters and nuclear receptors (Figs. 3d and 4a; Supplementary information, Fig. S3a). Immunofluorescence staining confirmed the protein expression of the major CYP450s in hiHeps (Fig. 4b; Supplementary information, Fig. S3b). In addition, the analysis of a large panel of drug metabolism-associated genes from RNA sequencing data revealed that hiHeps established a complex drug-metabolizing gene regulatory network (Fig. 3f).

Next, we performed ultra-performance liquid chromatography-tandem mass spectrometry (UPLC/MS/MS) to examine the drug-metabolizing activities of hiHeps. We analyzed seven major drug-metabolizing CYP450s (CYP3A4, CYP2D6, CYP2C9, CYP2C19, CYP1A2, CYP2B6 and CYP2C8), which account for over 90% of CYP450-metabolized commercial drugs.^17,22^ Notably, these seven enzymes cover all of the CYP450s documented by the FDA for drug evaluation.^23^ We found that the metabolic activities of all these CYP450s in hiHeps were comparable to those in PHHs (Fig. 4c). In addition, we analyzed the activities of UGTs using the metabolic substrate 7-HC by UPLC/MS/MS to determine the phase II biotransformation properties. The activities of UGTs in hiHeps were also comparable to that in F-PHHs (Fig. 4d). Furthermore, hiHeps showed the potential for drug clearance prediction for typical drugs metabolized by major CYP450s and UGT2B7. Intrinsic clearance (CL_int_) of hiHeps was scaled into in vivo hepatic clearance (CL_h_), and was comparable to the observed in vivo CL_h_ in previous reports (Supplementary information, Fig. S3c).^24,25^

To determine whether the activities of CYP450s were modulated by nuclear receptors in hiHeps, we exposed hiHeps to the agonists of drug metabolism-associated nuclear receptors. The PXR agonist (rifampin), AhR agonist (β-naphthoflavone) and CAR agonist (phenobarbital) were used to induce the activities of CYP3A4, CYP1A2 and CYP2B6, respectively. The results showed that these three CYP450s could respond to their corresponding inducers, and the levels of induction were comparable to that of PHHs (Supplementary information, Fig. S3d). In contrast, HepG2 cells showed no inducible activities to any of the agonists (Supplementary information, Fig. S3d). In addition, hiHeps responded to structurally different inducers of CYP3A4 (Supplementary information, Fig. S3e). Collectively, these data suggest that hiHeps have established an innate drug-metabolizing gene expression network and possessed the robust drug metabolic activities, which could replace PHHs for in vitro drug metabolism estimation.

### Comparable toxicity prediction ability between hiHeps and primary human hepatocytes

**Fig. 5:**
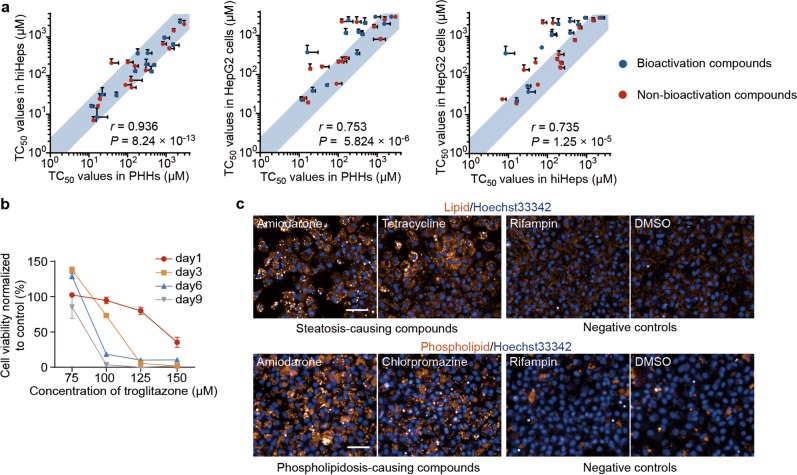
Comparable toxicity prediction ability of hiHeps to PHHs. **a** Comparison of the TC_50_ values in hiHeps, PHHs and HepG2 cells with 25 compounds. The light blue region represents a less than 2.5-fold difference of TC_50_ between compared cell types. *n* = 3 for all compounds in hiHeps, PHHs and HepG2 cells, except that *n* = 2 for AFB1 in PHHs. The correlation between groups was evaluated by the Pearson correlation coefficient (*r*). **b** Time- and dose-dependent chronic toxicity of troglitazone in hiHeps. Mitochondrial activities were normalized to DMSO-treated cultures at corresponding day. *n* = 6. **c** Fluorescence evaluation of the pathological effects of DILI in hiHeps. Steatosis (up) and phospholipidosis (down) were evaluated in hiHeps after exposure to steatosis-causing compounds and phospholipidosis-causing compounds, respectively. Rifampin (non-steatosis/phospholipidosis-causing drug) or DMSO treatment were set as controls. For all compounds, images were captured at the concentration of 80% TC_50_. For all measurements, ‘*n*’ represents the number of biological replicates. The scale bars represent 50 μm. Data are presented as mean ± SEM

In another series of experiments, we showed that hiHeps provided a powerful in vitro system for predicting liver drug toxicity as PHHs. First, we tested the hepatotoxicity of 25 compounds on hiHeps, HepG2 cells and PHHs for comparison. Among the 25 compounds, 23 compounds are FDA-approved compounds with drug-induced liver injury (DILI) and 2 compounds are common hepatotoxins.^26,27^ Compound hepatotoxicity was characterized by TC_50_, the concentration that results in 50% reduction of cell viability (Supplementary information, Fig. S4a). Notably, for all these 25 compounds, TC_50_ profiles of hiHeps were not discriminated from those of PHHs. In Contrast, HepG2 cells showed distinct TC_50_ profiles from hiHeps and PHHs (Fig. 5a). Among these drugs, bioactivation compounds, which were metabolized by hepatocytes into toxic products, showed lower TC_50_ profiles in hiHeps and PHHs than in HepG2 cells, consistent with the robust drug-metabolizing activity of hiHeps and PHHs (Fig. 5a). We tested the dose and time-dependent chronic toxicities of troglitazone, a representative chronic hepatotoxin.^28^ The non-lethal concentrations of troglitazone caused extensive cell death after prolonged 9 days of drug exposure, in line with previous studies on PHHs (Fig. 5b).^28^ Collectively, these results suggested that hiHeps showed an analogous function to PHHs for the evaluation of the acute and chronic hepatotoxicity.

We next tested whether drug-induced pathological effects could be recapitulated by hiHeps. The severe and dose-dependent steatosis and phospholipidosis were detected in hiHeps upon exposure to pathology-causing hepatotoxins, but not to non-pathology-causing hepatotoxin or the vehicle control (Fig. 5c; Supplementary information, Fig. S4b, c). Furthermore, we found that hiHeps could also be applied to evaluate toxicity caused by drug-drug interactions (DDIs). The toxicity of two bioactivation drugs, aflatoxin B1 (AFB1) and flutamide, increased after treatment with CYP3A4 inducer (rifampin) in hiHeps, and hiHeps were further rescued by CYP3A4 inhibitor (ketoconazole) (Supplementary information, Fig. S4d, f). In contrast, these DDI effects were not detected in HepG2 cells (Supplementary information, Fig. S4e, g). These results collectively indicate that hiHeps could replace PHHs for hepatotoxicity evaluation.

### hiHeps support long-term hepatitis B virus (HBV) infection in vitro

**Fig. 6:**
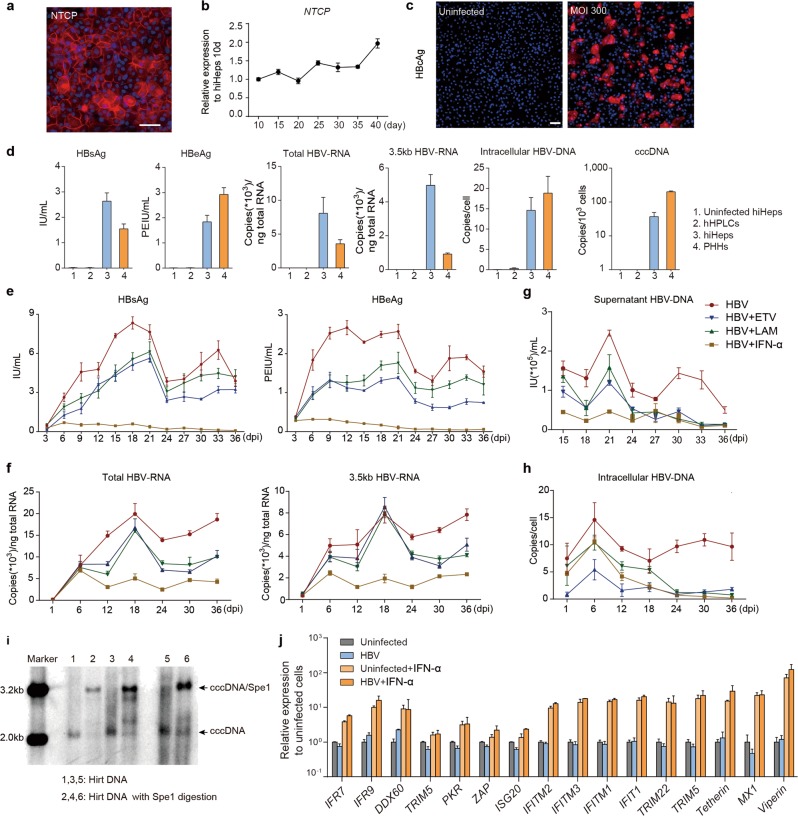
hiHeps are validated for in vitro disease modeling of HBV infection. **a** Immunofluorescence staining of NTCP in hiHeps. **b** Dynamic gene expression analysis of *NTCP* in hiHeps cultured in HMM for 40 days by RT-qPCR. *n* = 3. Relative expression was normalized to hiHeps cultured for 10 days. **c** Immunofluorescence staining of HBcAg in hiHeps infected with HBV at a multiplicity of infection (MOI) of 300 at 20 dpi and in uninfected hiHeps. **d** Quantification of different HBV markers in hHPLCs, hiHeps and PHHs around 6 dpi and in uninfected hiHeps. *n* = 3 **e**-**h** Dynamic expression of different HBV markers in 36 days post infection. HBV proteins (**e**), HBV-RNA (**f**), supernatant HBV-DNA (**g**) and intracellular HBV-DNA (**h**) were analyzed in HBV-infected hiHeps and hiHeps treated with ETV, LAM and IFN-α. *n* = 3. **i** Southern blot analysis of cccDNA in hiHeps. Hirt DNA was extracted from three independent experiments and half of the Hirt DNA was treated with *Spe*I before southern blotting. **j** Gene expression analysis of key ISGs in HBV-infected hiHeps, HBV-infected hiHeps treated with IFN-α, uninfected hiHeps and uninfected hiHeps treated with IFN-α. *n* = 3. For all measurements, ‘*n*’ represents the number of biological replicates. The scale bars represent 50 μm. Data are presented as mean ± SEM

Our recent study reported that primary hepatocytes could support long-term HBV infection,^18^ which however has limitations for large-scale anti-virus drug screening. Considering that hiHeps obtained the mature functions of hepatocytes and could be long-term maintained in culture, we tested whether hiHeps could serve as an in vitro model for HBV infection. First, we detected the expression of HBV receptor, NTCP, in hiHeps by RT-qPCR and immunofluorescence staining, and we found that NTCP was expressed at a comparable level to that of PHHs (Fig. 6a, b; Supplementary information, Fig. S3a). We next infected hiHeps with HBV, and successful infection was indicated by HBcAg-positive cells in hiHeps culture (Fig. 6c). To test the HBV propagation in hiHeps, we further analyzed the HBV products in hiHeps, including the secreted HBV antigens (HBsAg and HBeAg), and intracellular HBV-DNA, HBV-RNA (total HBV-specific transcripts and 3.5 kb transcripts) and covalently closed circular DNA (cccDNA). These HBV products in infected hiHeps were comparable to those in infected PHHs, but were undetectable in HBV-infected hHPLCs and uninfected hiHeps (Fig. 6d). Through dynamic monitoring of the HBV infection over 36 days, the secretion of HBsAg and HBeAg gradually increased and peaked at 20 dpi, followed by a plateau, which was correlated with the dynamic expression pattern of HBV-RNA (Fig. 6e, f). The secreted and intracellular HBV-DNA retained their expression for 36 days (Fig. 6g, h). Notably, the generation of cccDNA in HBV-infected hiHeps was detected by Southern blot (Fig. 6i). These results collectively indicate that hiHeps could efficiently support long-term HBV infection in vitro.

We then tested whether the hiHeps-based HBV model could be used for evaluating the antiviral drugs. To this end, we tested the response of hiHeps to two major classes of anti-HBV drugs, nucleoside analogs and interferons.^29^ The results showed that the nucleoside analogs entecavir (ETV) and lamivudine (LAM) greatly inhibited HBV-DNA, but showed a moderate effect on HBV-RNA and HBV-proteins (Fig. 6e–h). In particular, interferon-alpha (IFN-α) showed inhibitory effect on all major HBV markers stated above, which could be associated with the upregulation of many IFN-stimulated genes, especially antiviral effectors (Fig. 6e–h, j). This suggested an innate antiviral immune response in hiHeps upon IFN-α treatment. Collectively, these results indicate that hiHeps are of great value for anti-HBV drug screening.

### Robust generation of large amounts of functional hepatocytes for large-scale application

To fulfill the cell quantity demand for large-scale applications of hiHeps, we tested whether large amounts of functional hepatocytes could be generated from hHPLCs. We first evaluated the long-term expansion capacity of hHPLCs in vitro by continuously passaging hHPLCs at a 1:5 ratio and found that the population doubling time was similar between P10 and P30 (Fig. 1i). During passaging to P30, most cells remained a normal karyotype, and only 6% cells of 1 hHPLC cell line showed aneuploidy at P30 (Supplementary information, Fig. S1h). Furthermore, the global gene expression profiles of hHPLCs of every 10 passages up to P40 did not show significant change, suggesting transcriptomic stability of hHPLCs during in vitro expansion (Fig. 1g, h). These results indicated that hHPLCs could be stably expanded ~9 × 10^27^ folds in 40 passages. Remarkably, functional hiHeps were stably generated from both early and late passages of hHPLCs and showed similar gene expression profiles and hepatocyte functions by a systematic comparison (Supplementary information, Fig. S5a). Additionally, hHPLCs also showed stable gene expression and differentiation capability before and after cryopreservation (Supplementary information, Fig. S5b, c). Collectively, these results demonstrate that large amounts of functionally mature human hepatocytes can be generated from hHPLCs, which hold great promise for industrial applications of hiHeps.

We next tested whether functionally mature hiHeps could be generated from hHPLCs derived from different genetic backgrounds. An additional 4 hHPLCs cell lines were successfully established from fibroblasts of 3 different donors and one commercialized human neonatal foreskin fibroblast (HFF) line CRL-2097. Notably, all of these 4 hHPLC lines gave rise to functional hiHeps indicated by the similar global gene expression profiles by hierarchical clustering and principle component analysis (Supplementary information, Fig. S6a, b). In particular, hiHeps derived from CRL-2097 were fully characterized for typical hepatocyte-polygonal morphology, hepatic gene expression and functions (Supplementary information, Fig. S6c–g). Collectively, these results suggest the two-step reprogramming strategy as an effective path to generate abundant functional hepatocytes across different genetic backgrounds.

## Discussion

In this study, we developed a two-step lineage reprogramming strategy to generate large quantities of functionally competent hepatocytes in vitro via an expandable intermediate progenitor stage. The robust expansion capacity of the induced hepatic progenitors allowed the generation of induced hepatocytes in large amounts. Systematic characterization revealed that induced hepatocytes recapitulated the mature characteristics of primary human hepatocytes, making it feasible to utilize these cells in a wide range of applications, such as drug discovery and liver disease modeling.

The generation of functionally competent hiHeps lies in the generation of proliferative hepatic progenitors during the two-step lineage reprogramming, which was generated by combining genetic and chemical approaches. The induction of the hepatic progenitor fate in fibroblasts was initiated by a new 5-TF combination, which was identified through a 4-TFs+1 screening (Supplementary information, Fig. S1b). In addition to *GATA4*, *FOXA2*, *HNF4A* and *HNF6A*, *HHEX* was discovered as a new reprogramming factor essential for inducing hepatic progenitors (Supplementary information, Fig. S1b, f), which plays an important role in coordinating ventral/dorsal patterning in the posterior foregut endoderm towards hepatic endoderm during liver organogenesis.^30^ After 5-TFs induced hepatic progenitor conversion, we further developed a hepatic expansion medium using small molecules and growth factor (Fig. 1b, c), which promoted not only the proliferation of reprogrammed cells but also the establishment of hepatic progenitor identity (Fig. 1e, i). Notably, the employment of all 5 exogenous factors and hepatic expansion medium is required for generating hepatic progenitors with a high expandable capacity (Fig. 1b; Supplementary information, Fig. S1f), suggesting a synergistic effect between these two entities in inducing a proliferative hepatic progenitor state.

The most important feature on the applied side of our work is the generation of abundant and functionally competent hepatocytes through robust hHPLC expansion that are highly amenable for large-scale in vitro applications of human hepatocytes. PHHs have applicative potential in vitro and our newly developed 5C condition permits their long-term functional maintenance in vitro.^18^ However, the limited source of and restricted accessibility to PHHs make them difficult to be applied in large-scale and high-throughput applications. Although recent studies have shown in-vitro expansion of primary hepatocytes under certain conditions,^31–35^ the batch-to-batch variation of PHHs potentially affects the functional maintenance and proliferation of PHHs in vitro. Moreover, the proliferative ability of primary hepatocytes is limited as late-passage expanding primary hepatocytes displayed reduced proliferative capacity.^35^ Compared to a recent study that showed an ~7.5 × 10^4^ fold expansion of primary hepatocytes in 30 days,^35^ our hHPLCs could stably expand ~10^6^ fold in 30 days, higher by orders of magnitude. Furthermore, our hHPLCs could stably expand for more than 40 passages (Fig. 1g–i), thus theoretically producing ~9 × 10^27^ mature hepatocytes from one progenitor cell, which is sufficient for large-scale applications. Moreover, hepatic progenitors at early or late passages could produce hiHeps at similar efficiency (Fig. 3b). Furthermore, hiHeps derived from early and late passages of hepatic progenitors were similar in terms of morphology, gene expression patterns and functionality (Supplementary information, Fig. S5a). These data suggested that hHPLCs and hiHeps generated by our strategy are promising for being standardized for large-scale applications. Therefore, our hepatic progenitors could serve as a stable source to generate abundant hepatocytes and meet the quantitative demand of industrial applications of human hepatocytes in vitro.

A key functional feature of hHPLCs is their robust ability to repopulate mouse liver (Fig. 2a, b). Importantly, in the mouse liver, hHPLC-derived human hepatocytes expressed a series of CYP450 enzymes (Fig. 2d), which account for the metabolism of more than 80% of marketed drugs.^17^ Accordingly, this data suggest that hHPLCs may engraft and generate human hepatocytes with metabolizing functions in vivo. Another important finding is that NTCP expression was robustly observed in hHPLC-derived hepatocytes (Fig. 2d), suggesting that these cells might be useful as a model of HBV infection in vivo. These data highlight hHPLCs as a promising new cell resource for developing humanized liver mouse models, which have been extensively used in establishment of human hepatitis virus infection models and drug metabolism studies. Currently the application of humanized liver mouse models is restricted by the shortage of primary human hepatocytes. The high in-vitro expansion ability of hHPLCs permits producing large amounts of hepatic cells with robust engrafting ability, sufficient to meet the needs of generating humanized liver mouse models in large-scale. Therefore, the application of hHPLCs would significantly advance the availability of humanized liver mouse models in the future.

Importantly, to obtain functionally competent hepatocytes at the second step during the two-step reprogramming, it is pivotal to devise the culture conditions to promote terminal differentiation of hHPLCs. The generation of functionally mature cells in vitro has been hindered by the lack of an efficient environment to boost cells to a terminally differentiated state. Our hepatocyte maturation medium was developed based on conditions that we designed for the long-term functional maintenance of primary human hepatocytes.^18^ Using this medium, hHPLCs rapidly lost progenitor features and acquired mature hepatic properties in terms of polygonal hepatocyte morphology, key hepatic marker expression and basic hepatic functions (Fig. 3). Moreover, the global transcriptional profile of hiHeps highly resemble that of freshly isolated PHHs (Fig. 3e, f), further suggesting maturity of hiHeps. The acquisition of a mature state by hiHeps in the hepatocyte maturation medium suggested that a culture condition maintaining primary adult cells permitted in-vitro generated cells to capture their terminally differentiated state, which would fit for other contexts, such as differentiation of functional cells from pluripotent stem cells.

Finally, our in vitro**-**generated hiHeps efficiently recapitulated mature hepatocyte functionality, particularly robust drug metabolic ability. Drug-metabolism capacity via CYP450 enzymes is a crucial feature of mature hepatocytes.^36^ In each new drug application, the FDA typically requires a statement on the relationship of the chemical entity under review to the CYP450s.^23^ In our hiHeps, all the FDA-documented CYP450s for drug discovery, whose functions cover the oxidation of over 90% of human drugs,^17,22^ were functionally validated in hiHeps, which were comparable to those in adult primary hepatocytes (Figs. 3f and 4a, c). Notably, the functionality of these CYP450s in those hepatocytes generated in vitro was not achieved in previous studies.^7,8,19–21^ Moreover, hiHeps could respond to various inducers, which further enables their applications in induction and drug-drug interaction screenings (Supplementary information, Fig. S3d, e).^17^ In addition, we also showed that hiHeps could be used to evaluate acute and chronic hepatotoxicity and support persistent HBV infection for over a month in vitro with robust generation of HBV products (Figs. 5 and 6). These data strongly highlight the utility of hiHeps in place of PHHs in drug development and disease modeling in large-scale applications.

In summary, we demonstrate a two-step reprogramming strategy that provides an effective route to generate a large amount of functional competent hepatocytes in vitro. The combination of the genetic and chemical approaches enables inducing a plastic proliferative progenitor stage during reprogramming, which promotes cell-fate conversion through overcoming reprogramming barriers and permits the large-scale generation of desired functional cells for application. Similar strategies may become a general path to generate other mature cell types in vitro.

## Materials and methods

### Human primary cell isolation and cell culture

The present study was approved by the Clinical Research Ethics Committee of China-Japan Friendship Hospital (Ethical approval No: 2009–50) and Stem Cell Research Oversight of Peking University (SCRO201103-03), and it was conducted according to the principles of the Declaration of Helsinki.

Human embryonic skin was obtained from aborted tissue with informed patient consent, and detailed information was listed in Supplementary information, Table S3. Human embryonic skin tissues were minced with forceps and incubated in 1 mg/mL collagenase IV (Gibco) for 1–2 h at 37 °C. After enzyme treatment, cells were collected by centrifugation and resuspended in HEF medium (Dulbecco’s modified Eagle’s medium (DMEM, Gibco) containing 10% fetal bovine serum (FBS, Ausbian), 1% GlutaMAX (Gibco), 1% Non-Essential Amino Acids (NEAA, Gibco) and 1% penicillin/streptomycin (PS, Gibco)). Cells were plated on 10 cm tissue culture dishes and cultured in HEF medium.

Fetal liver tissues were obtained from aborted tissue with informed patient consent. To obtain fetal liver cells, fetal liver tissues were cut into 1–3 mm^3^ fragments for digestion in 10 mL of RPMI 1640 medium supplemented with 1 mg/mL collagenase IV. Digestion was performed at 37 °C for 15–20 min, and erythrocytes were eliminated by low-speed centrifugation. Cells were washed 3 times with RPMI 1640 medium and collected by centrifugation.

Primary human hepatocytes (PHHs) were isolated from human donor livers with informed consent. Detailed information were listed in Supplementary information, Table S4. Briefly, liver tissues were perfused with collagenase IV and dispase (Sigma-Aldrich) until the tissues were incompact and separated with tweezers. Hepatocytes were washed 3 times with HCM^TM^ (Lonza), plated in collagen-coated plates and cultured in HCM^TM^. For experiments longer than 3 days, PHHs were cultured in William’s E medium containing 2% B27 (Gibco), 1% GlutaMAX, and 50 μM forskolin and 10 μM SB431542. For sandwich culture experiment, PHHs were plated and overlaid with ice-cold 0.25 mg/mL Matrigel (BD Biosciences) in DMEM supplemented with 1% ITS (Gibco), 1% GlutaMAX, 1% NEAA, 1% PS and 10^−7^ M dexamethasone for 24 h. Cell culture and further experiments were all processed in this medium.

HepG2 cell line was a gift from Kuanhui Xiang (Peking University Health Science Center) and cultured in DMEM containing 10% FBS, 1% GlutaMAX, 1% PS and 1% NEAA (Gibco).

### Molecular cloning and lentivirus production

Complementary DNA of the five hepatic transcription factors were amplified from human full-length TrueClones™ (Origene) and inserted into pCDH-EF1-MCS-T2A-Puro (System Biosciences) according to the user’s manual. *c-MYC* was cloned into an inducible system of Fu-tet-hOct4 (hOct4 was replaced by c-MYC).^37^ FUdeltaGW-rtTA was from Addgene. The oligonucleotides encoding p53 siRNA were 5′-TGACTCCAGTGGTAATCTACTTCAAGAGAGTAGATTACCACTGGAG TCTTTTTTC-3′ and 5′-TCGAGAAAAAAGACTCCAGTGGTAATCTACTCTCTT GAAGTAGATTACCACTGGAGTCA-3′. The oligonucleotides were ligated downstream of the U6 promoter in a Lenti-Lox3.7 (pLL3.7) vector.^38^ Lentivirus production and collection were described previously.^38^

### Generation of hHPLCs and hiHeps

Human fibroblasts used for hepatic conversion is at passage 2 to passage 6 and were plated at the density of approximate 10^4^ cells per well in 12-well plates (Falcon) on the day before infection. Fibroblasts were infected with lentivirus carrying the 5 hepatic transcription factors, c-MYC, rtTA and P53 siRNA at a multiplicity of infection (MOI) around 15 in HEF medium containing 10 μg/mL polybrene. The titre of each virus was calculated by a qPCR Lentivirus Titration Kit (abm) according to the manufacturer’s instructions. After 12 h, cells were washed with PBS and cultured in HEF medium containing 2 µg/mL doxycycline for 7 days. During this period, infected cells proliferated and reprogrammed with epithelial clones emerging at around 7 dpi. The infected cells were treated with 2 μg/mL puromycin for 24 h at 7 dpi and then splitted with Accutase (Gibco) at a ratio of 1:3–4 according to the cell density. Afterward, reprogrammed cells were cultured on cell culture plates coated with 0.2 mg/mL Matrigel. During cell passaging, a fraction of infected cells was harvested for RNA extraction for RT-qPCR analysis to check the expression of the exogenous reprogramming factors. A secondary infection was conducted if some exogenous factors had failed to be overexpressed. The purpose of the secondary infection was to ensure that all exogenous factors were inserted into the genome, and the secondary infection could be performed after passage. After passage, cells were cultured in HEM (50% DMEM/F12 and 50% William's E Medium supplemented with 1% PS, 2% B27 (without V_A_), 5 mM Nicotinamide, 200 μM 2-phospho-L-ascorbic acid (pVc), 3 μM CHIR99021, 5 μM SB431542, 0.5 μM Sphingosine-1-phosphate (S1P), 5 μM Lysophosphatidic acid (LPA), 50 ng/mL EGF and 2 µg/mL doxycycline). The reprogramming cells are cultured in HEM for 20–30 days, and passaged at a ratio of 1:3 every 5 days. Reprogrammed cells were indicated as hHPLCs based on the criteria in order of completed epithelial conversion of whole-well cells, hepatic progenitor gene expression, and response to the maturation medium to produce functional hepatocytes. hHPLCs were maintained in HEM and passaged every 5 days at a ratio of 1:5.

To generate functional hiHeps, hHPLCs were cultured until compactly confluent on 0.2 mg/mL Matrigel-coated plates and were then treated with hepatocyte maturation medium (HMM (HCM^TM^ minus EGF plus 50 μM forskolin or 50 μM dbCAMP and 10 μM SB431542)) for 7–10 days.

### Gene expression analysis

Total RNA was isolated by Direct-zol RNA Miniprep (ZYMO RESEARCH) and then reverse-transcribed with TransScript First-Strand cDNA Synthesis SuperMix (TransGen Biotech). RT-qPCR was performed using KAPA SYBR® FAST Universal qPCR Mix (KAPA Biosystems) on a BIO-RAD CFX384^TM^ Real-time System. The quantified values were normalized against the input determined by housekeeping genes (*RPL13A* or *RRN18S*). The RT-qPCR primer sequences were provided in Supplementary information, Table S5.

### Immunofluorescence staining

Cells were fixed in 4% paraformaldehyde (PFA; DingGuo) at room temperature for 15 min and blocked with PBS containing 0.25% Triton X-100 and 5% normal donkey serum (Jackson ImmunoResearch Laboratories, Inc.) at room temperature for 1 h. Mouse liver tissues were fixed in 4% paraformaldehyde for 2 h at room temperature and dehydrated with 30% sucrose solution over night before embedded in OCT compound (Sakura) for cryosectioning. Samples for immunofluorescence staining were incubated with primary antibodies at 4 °C overnight, washed three times with PBS and then incubated with appropriate secondary antibodies for 1 h at room temperature in the dark. Nuclei were stained with DAPI (Roche). The primary antibodies used for immunofluorescence were listed in Supplementary information, Table S6. The secondary antibodies used for immunofluorescence were as follows: DyLight® 550 Donkey anti rabbit, DyLight® 550 Donkey anti goat, DyLight® 550 Donkey anti mouse, DyLight® 650 Donkey anti goat, DyLight® 650 Donkey anti mouse and DyLight® 650 Donkey anti rabbit (all from Abcam). For quantification of ALB-positive cells, images were randomly taken at 10× and 20× magnification at the same exposure using an Operetta High-Content Imaging System (PerkinElmer) and then analyzed by Columbus Image Data Storage and Analysis System.

### Flow cytometry

Cell culture was dissociated into single-cell suspension by Accutase (Millipore) at 37 °C for 3–5 min. Cells were washed using basal medium. Thoroughly re-suspended cells were added in Fixation/Permeabilization solution (BD Biosciences) for 20 min at 4 °C, and washed twice in 1× BD Perm/Wash buffer. After that cells were incubated with conjugated primary antibodies in 200 µL Staining buffer consisting of Perm wash buffer with 2% normal goat serum in 4 °C for 2 h. Stained cells were washed twice in BD Perm/Wash buffer and incubated in 200 µL staining buffer containing secondary antibodies. After washed twice in BD Perm/Wash buffer, cells were re-suspended in BD Perm/Wash buffer and analyzed on BD FACSCalibur flow cytometry system. The data were analyzed by FlowJo software.

### Albumin ELISA, PAS staining, LDL uptake and oil red O staining

Secretion of human albumin was measured using the Human Albumin ELISA Quantitation kit (Bethyl Laboratory) according to the manufacturer’s instructions. The PAS staining system was purchased from Sigma-Aldrich. Cultures were fixed with 4% paraformaldehyde (DingGuo) and stained according to the manufacturer’s instructions. For the LDL uptake assay, hiHeps were incubated with 10 μg/mL DiI-Ac-LDL (Invitrogen) for 4 h and 1 μg/mL Hoechst 33342 (Thermo Fisher Scientific) for 30 min at 37 °C and then washed 3 times before imaging using fluorescence microscopy. Lipid detection was performed with a Lipid (Oil Red O) Staining Kit (Sigma) according to the manufacturer’s instructions.

### Animals and transplantation

Tet-uPA/Rag2^–/–^/γc^–/–^ (URG) mice on a BALB/c background were purchased from Beijing Vitalstar Biotechnology. Cells for transplantation were suspended into single cells in HCM^TM^ medium. 2 × 10^6^ cells in 200 μL suspension were injected into the spleen of the mice. Blood samples were collected and human albumin was quantified using the Human Albumin ELISA Quantitation kit (Bethyl Laboratories). Livers of recipient mice were fixed with 4% paraformaldehyde and dehydrated with 30% sucrose solution followed by embedded in OCT compound (Sakura) and frozen in liquid nitrogen. Cryo-sections were generated using cryostat (Leica) and were further stained as described in the method of immunofluorescent staining. Images were captured with Vectra Polris (PerkinElmer). The repopulation rate of each humanized mouse liver was calculated using at least 12 random sections, and the hALB^+^ regions were identified as the repopulation region.

NOD-Prkdc^scid^ Il2rg^null^ (NPG) mice were used as tumour-bearing mice and were purchased from Beijing Vitalstar Biotechnology. Cells were collected and suspended in Matirgel at a concentration of 2 × 10^7^ cells/mL. 50 μL of cell suspension were subcutaneously injected into the left and right sides of NPG mouse. Transplantation of HepG2 cells led to the formation of tumor at 2 to 3 weeks post-transplantation and the mice were sacrificed for further analysis.

All the animal procedures were performed according to NIH guidelines, and the mouse work performed were approved by the Institutional Animal Care and Use Committee of Peking University.

### Measurements of cytochrome P450 and UDP-glucuronosyltransferase (UGT) activities

The methods for measuring the CYP450 activity were described previously.^8^ Briefly, hiHeps and HepG2 cells were dissociated and suspended to measure their CYP450 or UGT activities. The drug metabolic activities of F-PHHs were measured immediately after isolation. Commercial cryopreserved PHHs were purchased from RILD (Shanghai) and used to detect drug metabolic activities immediately after resuscitation. One 500 μL reaction contained 2.5 × 10^5^ cells and the indicated substrates. After incubation for 15–30 min at 37 °C in an orbital shaker, the reactions were stopped by the addition of sample aliquots to tubes containing triple the volume of quenching solvent (methanol) and were frozen at –80 °C. Isotope-labeled reference metabolites were used as internal standards for further mass spectrometry (ultra-performance liquid chromatography-tandem mass spectrometry, UPLC/MS/MS) analysis. The details of the substrates, internal standards and other relevant information of CYP450 metabolism were listed in Supplementary information, Table S7. Conjugation of 7-Hydroxycoumarin with glucuronic acid groups is mediated by Phase II enzymes (UDP-glucuronosyltransferase). The substrate of Phase II enzyme is 100 μM of 7-Hydroxycoumarin. The product of this reaction is 7-Hydroxycoumarin Glucuronide, and the internal standards is Rutin. UPLC/MS/MS analyses were performed using an ACQUITY H-Class UPLC System (Waters) coupled to a Sciex API4500Q-trap Mass Spectrometer (SCIEX). The analytical column was an ACQUITY UPLC® BEH C18 1.7 μm 2.1 × 50 mm (CYP450s activity) or ACQUITY UPLC® HSS T3 1.8 μm 2.1 × 100 mm (Phase II activity) coupled with a preguard column. The results were presented as picomoles of metabolite formed per minute and per million cells. hiHeps matured in HMM were used to measure the activity of CYP3A4, CYP2B6, CYP2C8, CYP2D6 and Phase II enzyme activities. To measure the activity of CYP1A2, hiHeps were generated in HMM with additional 3 μM CHIR-99021 and 2 μM U0126 for 10 days. To measure the activities of CYP2C9 and CYP2C19, hiHeps were matured in HMM and further cultured in HCM^TM^ plus 10 μM rifampin for 3 days.

To measure the induced activity of CYP3A4, CYP2B6 and CYP1A2, hiHeps matured in HMM were further cultured for induction in HCM^TM^ with 50 μM rifampin, 1 mM phenobarbital and 50 μM β-naphthoflavone for 3 days, respectively. Vehicle-treated groups were used to determine the basal activity. To measure the induced activity of PHHs, PHHs were cultured under sandwich condition immediately after isolation and incubated with inducers listed above or vehicle for three days.

### Measurements of hepatic clearance

Measurements of clearance were performed as previously described.^24,39^ Briefly, a 1 × 10^6^ cells/mL cell suspension and a 2× drug solution were prepared in incubation medium (William’s E medium, 10 mM HEPES [pH 7.4], and 1% GlutaMAX). Reactions were started by adding 500 μL of drug solutions to 500 μL of hiHeps, giving a final substrate concentration of between 1 and 2 μM. The detailed information of the substrates is listed in Supplementary information, Table S8. These concentrations were selected to be below the K_m_ for most substrates but to still have sufficient analytical sensitivity. Reactions were performed in an orbital shaker in an incubator at 37 °C. Then, 80 μL aliquots were removed at 0, 15, 30, 60, 90, 120, 180 and 240 min, and samples were quenched in 240 μL of methanol containing an isotope-labeled reference and frozen at −80 °C. The substrates were quantified using the validated traditional UPLC-MS/MS methods described above. Assays were performed in triplicate.

In vitro intrinsic clearance (CL_int_) was determined using the rate of parent disappearance as described previously.^39,40^ The slope (−k) of the linear regression from the log [substrate] versus time plot was determined. Because the elimination rate constant was k = 0.693/t_1/2_, an equation expressing CL_int_ in terms of t_1/2_ of parent loss was derived as follows: Cl_int = _volume × 0.693/t_1/2_. The hepatocyte CL_int_ (units, μL/min/10^6^ cells) was scaled to the in vivo CL_int_ (units, mL/min/kg) using the following physiological parameters: human liver weight of 22 g/kg body weight and hepatocellularity of 120 × 10^6^ cells/g of liver. The projection of human in vivo hepatic clearance (CL_h_) was made using an adapted version of the nonrestrictive well stirred model as follows:^41^ CL_h = _(CL_int_ × Q_h_)/(CL_int_ × Q_h_), where Q_h_ is hepatic blood flow (human Q_h_ = 20 mL/min/kg). No correction factor was made for any differential in vitro and in vivo binding, and the distribution of the drug between the plasma and blood was assumed to be unity.

### Toxicity assay

For the toxicity assay, hiHeps, HepG2 cells and PHHs were cultured in 96-well or 384-well plates. Compounds with 7–8 concentration dilutions were prepared in DMEM with 3% FBS for HepG2 cells or in HCM^TM^ for PHHs and hiHeps. The final DMSO concentration under all conditions was consistent. The detailed information of the tested compounds was listed in Supplementary information, Table S9.^26,27^ Compounds were tested in bipartite in a dilution series by half-log concentration increments. After the cells were treated with compounds for 24 h, the supernatant was discarded and refreshed with modified WEM (William’s E medium supplemented with 2% B27 and 1% GlutaMAX) containing fluorescent probes. The following three fluorescent probes were simultaneously used to monitor cells in culture: 2 μM CellTrace™ Calcein Red-Orange AM (Thermo Fisher Scientific), 0.1 μM MitoTracker™ Deep Red FM (Thermo Fisher Scientific) and 1 μg/mL Hoechst 33342 (Thermo Fisher Scientific). After incubation for 30 min, the supernatant was discarded and cells were washed twice. Images were acquired using the Operetta High-Content Imaging System (PerkinElmer) with a 10× objective. The supernatant containing fluorescent probes was discarded, and cells were further incubated with modified WEM containing 9.1% CCK-8 (Dojindo). After chromogenic reaction for 1–4 h in the incubator, the absorbance at 450 nm was read using a SpectraMax i3x (Molecular Devices). Image data were analyzed online using the Columbus Image Data Storage and Analysis System (PerkinElmer) with the following parameters: (1) identification and counting of nuclei; (2) identification and counting of live cells labeled by CellTrace™ Calcein Red-Orange AM; and (3) identification and counting of live cells labeled by MitoTracker™ Deep Red FM. The viability of three parameters described above were transformed from image data, and the CCK-8 assay data, which is the fourth parameter, were entered into Excel. The cellular viability of each parameter was expressed as the live cell ratio and normalized to the negative control. The TC_50_ values, which is the concentration that results in 50% reduction of cell viability (Supplementary information, Fig. S4a), of these four parameters were calculated separately, and the minimum TC_50_ value was used as the final TC_50_.

### Steatosis and phospholipidosis assay

Imaging and quantification of intracellular lipids were performed using HCS LipidTOX™ Deep Red Neutral Lipid Stain (1000×) (Thermo Fisher Scientific) according to the manufacturer’s directions. Briefly, hiHeps were incubated with compounds with the following concentration gradient: 100%, 80%, 60%, 40%, 20 and 0% TC_50_. The TC_50_ vales of amiodarone, tetracycline hydrochloride and rifampin were 30 μM, 400 μM and 100 μM, respectively, which were all rounded for ease of use. The final DMSO concentration of all of the tested wells and control wells was 0.1%. After incubation with compounds for 24 h, cells were fixed with 4% PFA. Nuclei were stained with DAPI, and lipids were stained with 1× Neutral Lipid Stain. Images were captured using an Operetta High-Content Imaging System (PerkinElmer) and analyzed with Columbus Image Data Storage and Analysis System.

Quantification of intracellular phospholipids was performed using HCS LipidTOX™ Red Phospholipidosis Detection Reagent (1000×) (Thermo Fisher Scientific) according to the manufacturer’s instructions. Briefly, hiHeps were cultured at a final concentration of 1× Phospholipidosis Detection Reagent and the following tested compounds: amiodarone (TC_50_ ≈ 30 μM), chlorpromazine (TC_50_ ≈ 25 μM) and rifampin (TC_50_ ≈ 100 μM). The TC_50_ values were all rounded for ease of use. Compounds were tested in the following concentration gradient: 80%, 60%, 40%, 20 and 0% TC_50_. The final DMSO concentration of all of the tested wells was consistent. After incubation with the compounds and detection reagent for 24 h, cells were fixed with 4% PFA, and nuclei were stained with DAPI. Images were captured using an Operetta High-Content Imaging System and analyzed with Columbus Image Data Storage and Analysis System.

### Drug-drug interaction assay

hiHeps in the DMSO group were cultured in HCM^TM^ supplemented with DMSO for 3 days. hiHeps in the rifampin (RIF) group were cultured in HCM^TM^ supplemented with 20 μM rifampin for 3 days. hiHeps in the RIF + KC (ketoconazole) group were cultured in HCM^TM^ supplemented with 20 μM rifampin for 3 days, and KC was added on the third day. The final DMSO concentration of these conditions on each day was unified to the highest concentration among these three groups. Aflatoxin B1 and flutamide were further tested on these cells following the toxicity assay.

### HBV infection and analysis of HBV replication intermediates

HBV was concentrated from the supernatant of the HBV-producing HepAD38 cell line using centrifugal filter devices (Centricon Plus-70, Biomax 100.000, Millipore Corp., Bedford, MA) and titered by HBV-DNA using RT-qPCR (KHB, Shanghai, China). hiHeps and PHHs were infected with HepAD38-derived HBV at a MOI around 300 in HCM^TM^ containing 2% DMSO and 4% PEG 8000 (Sigma Aldrich) for 16–20 h. After infection, cells were washed 9 times with PBS and cultured in HMM supplemented with 1 μM DAPT, 0.5 μM IWP2 and 0.1 μM LDN193189.^18^ hHPLCs were infected in HEM containing 4% PEG 8000 and cultured in HEM. The medium was changed every 3 days, and supernatants were collected. For inhibition of the HBV life cycle, LAM (TargetMol) and ETV (TargetMol) were used at 1 μM and 0.5 μM, respectively, during and after infection. IFN-α (Genway) was used at 1000 U/mL after infection.

The HBV viral proteins HBsAg and HBeAg were examined using 50 μL of supernatants with commercial ELISA kits (Autobio, Henan, China) following the manufacturer’s instructions. Extracellular HBV DNA quantification was performed via DNA extraction using a HBV detection kit (KHB, Shanghai, China). Intracellular HBV-DNA was extracted using a DNeasy Blood & Tissue kit (QIAGEN) and quantified using the following specific primers by real-time PCR: 5′-GAGTGTGGATTCGCACTCC-3′ (forward) and 5′-GAGGCGAGGGAGTTCTTCT-3′ (backward).^42^ The viral genome equivalent copies were calculated based on a standard curve generated from samples with known copy numbers. Real-time PCR was performed using KAPA SYBR® FAST Universal qPCR Mix (KAPA Biosystems) and a BIO-RAD CFX384TM Real-time System.

Quantification of HBV-specific RNAs was performed as previously described.^42^ To quantify HBV-specific RNAs, total RNA was isolated from HBV-infected cells using the Direct-zol RNA Miniprep kit (ZYMO RESEARCH). Approximately 400 ng of total RNA was reverse transcribed into cDNA with TransScript First-Strand cDNA Synthesis SuperMix (TransGen Biotech) and equal total RNA was directly used as template to exclude the HBV viral DNA contamination in the RNA preparation. The following primers were used for the HBV 3.5-kb transcripts: 5′-GAGTGTGGATTCGCACTCC-3′ and 5-GAGGCGAGGGAGTTCTTCT-3′. The following primers were used for the total HBV-specific transcripts: 5′-TCACCAGCACCATGCAAC-3′ and 5′-AAGCCACCCAAGGCACAG-3′. Quantification of these reversed cDNA and total RNA was performed by real-time PCR.

Quantification of HBV cccDNA was performed using rolling circle amplification in combination with real-time PCR as previously described by Zhong et al.^43^ Single-stranded and relaxed circular DNAs were degraded before amplification by treating the DNA templates with plasmid-safe adenosine triphosphate (ATP)-dependent deoxyribonuclease DNase (PSAD, Epicentre Technologies). PSAD-treated samples were subjected to rolling circle amplification (RCA) prior to real-time PCR mediated by cccDNA-selective primers. Four pairs of primers were designed for mediating RCA:

**Table:** 

RCA1	AATCCTCACAATA *C*C	99–113
RCA2	ACCTATTCTCCTC *C*C	1758–1744
RCA3	CCTATGGGAGTGG *G*C	510–524
RCA4	CCTTTGTCCAAGG *G*C	2689–2675
RCA5	ATGCAACTTTTTC *A*C	1686–1700
RCA6	CTAGCAGAGCTTG *G*T	29–15
RCA7	TAGAAGAAGAACT *C*C	2240–2254
RCA8	GGGCCCACATATT *G*T	2599–2585

The reaction was performed with Phi29 DNA polymerase (New England Biolabs, Worcester, MA) at 30 °C for 16 h and terminated at 65 °C for 10 min. Using the RCA products as a template, HBV cccDNA was further amplified and quantified with real-time PCR mediated by a pair of cccDNA-selective primers (5′-GGGGCGCACCTCTCTTTA-3′ 1521-1538; 5′-AGGCACAGCTTGGAGGC-3′ 1886-1870).

For southern blot analysis of HBV cccDNA, we referenced the method provided by Cai et al. with modifications.^44^ Briefly, a modified Hirt method was used to extract proteins-free viral DNA as described, and half of the extracted DNA sample was treated with *Spe*I (NEB). For Southern blotting, the DNA was separated on a 1.2% agarose gel and then transferred to a Hybond-XL membrane. The 3.2 kb and 2.0 kb HBV DNA fragments were also run on the same agarose gel to serve as the molecular markers. Southern blot was performed with the DIG High Prime DNA Labeling and Detection Starter Kit II (Roche, 11 585 614 910), with reference to “Roche Techniques for Hybridization of DIG-labeled Probes to a Blot”. Lanes 1–4 were Hirt DNA from hiHeps infected with HBV from patient sera, and lanes 5–6 were Hirt DNA from hiHeps infected with HepAD38-derived HBV.

### RNA sequencing and bioinformatics analysis

RNA-Seq was performed at Hangzhou Repugene Technology Co., Ltd. Total RNA was isolated from HEFs, hHPLCs, hiHeps, hFLCs, HepG2 cells, F-PHHs and ALs using the RNeasyMini kit (QIAGEN). RNA sequencing libraries were prepared using the NEBNext UltraTM RNA Library Prep kit for Illumina (NEB, USA) following the manufacturer’s recommendations. The fragmented and randomly primed 150-bp paired-end libraries were sequenced on Illumina Hiseq 4000 platform. The generated sequencing reads were mapped against the human genome build hg19 using STAR, and the read counts for each gene were calculated using featureCounts. Gene expression was normalized by DESeq2, and the low expression genes with total counts across all samples less than 1 were excluded. Unsupervised hierarchical clustering of RNA-seq data was conducted by the hclust package in R (R 3.4.3, https://www.r-project.org). Heatmaps were generated by the pheatmap package (pheatmap 1.0.8, https://github.com/raivokolde/pheatmap).

### Growth curve and doubling times

The cell numbers of fibroblasts and hHPLCs at different passages were counted at days 0, 2, 3 and 4 after plating cells in 12-well plates. Given the measurements of the growing quantity, q_1_ (units, cell) at day 0 and q_2_ at time t_2_ (units, hour), the doubling time, T_d_ (units, hour), was calculated as follows:

$${\mathrm{T}}_{\mathrm{d}} = \left( {{\mathrm{t}}_2 \times \ln 2} \right)/\left( {\ln \left( {{\mathrm{q}}_2/{\mathrm{q}}_1} \right)} \right)$$

### Statistical analysis

The sample size was not predetermined by any statistical method and depended on the experiment type based on the standard practice in the field of lineage reprogramming and stem cell biology as well as on the basis of preliminary data. The experiments were not randomized, and the investigators were not blinded to the allocation during experiments and outcome assessment. For all measurements, ‘*n*’ represents the number of biological replicates. Experiments were independently replicated at least twice, and representative data are shown. *P* values for the purpose of group comparisons were calculated using one-way ANOVA. Correlations were evaluated using Pearson correlation coefficients. The level of significance in all graphs is represented as follows: *P < 0.05, **P < 0.01, ***P < 0.001. Unless described otherwise, standard statistical analyses were performed with GraphPad Prism 7 using default parameters. All of the error bars represent SEM.

### Code and data availability

The bioinformatics scripts used to analyze the data presented in the study will be available on GitHub (http://github.com/pmb59/neMeRIP-seq) before publish. The RNA sequencing data are available in the Gene Expression Omnibus (GEO) under the accession number GSE112330. All figures have associated raw data, and data supporting the conclusions of this study are available from the corresponding author upon reasonable request.

## Supplementary information

Supplementary information, Figure S1

Supplementary information, Figure S2

Supplementary information, Figure S3

Supplementary information, Figure S4

Supplementary information, Figure S5

Supplementary information, Figure S6

Supplementary information, Table S1

Supplementary information, Table S2

Supplementary information, Table S3

Supplementary information, Table S4

Supplementary information, Table S5

Supplementary information, Table S6

Supplementary information, Table S7

Supplementary information, Table S8

Supplementary information, Table S9
