# Supplementary material for: A two-step lineage reprogramming strategy to generate functionally competent human hepatocytes from fibroblasts
Source: Cell Res. 2019 Jul 3;29(9):696–710. doi: 10.1038/s41422-019-0196-x (PMC6796870; doi:10.1038/s41422-019-0196-x)
Supplement: Supplementary file 1 — Supplementary information, Figure S1 [file 41422_2019_196_MOESM1_ESM.pdf]

Figure S1

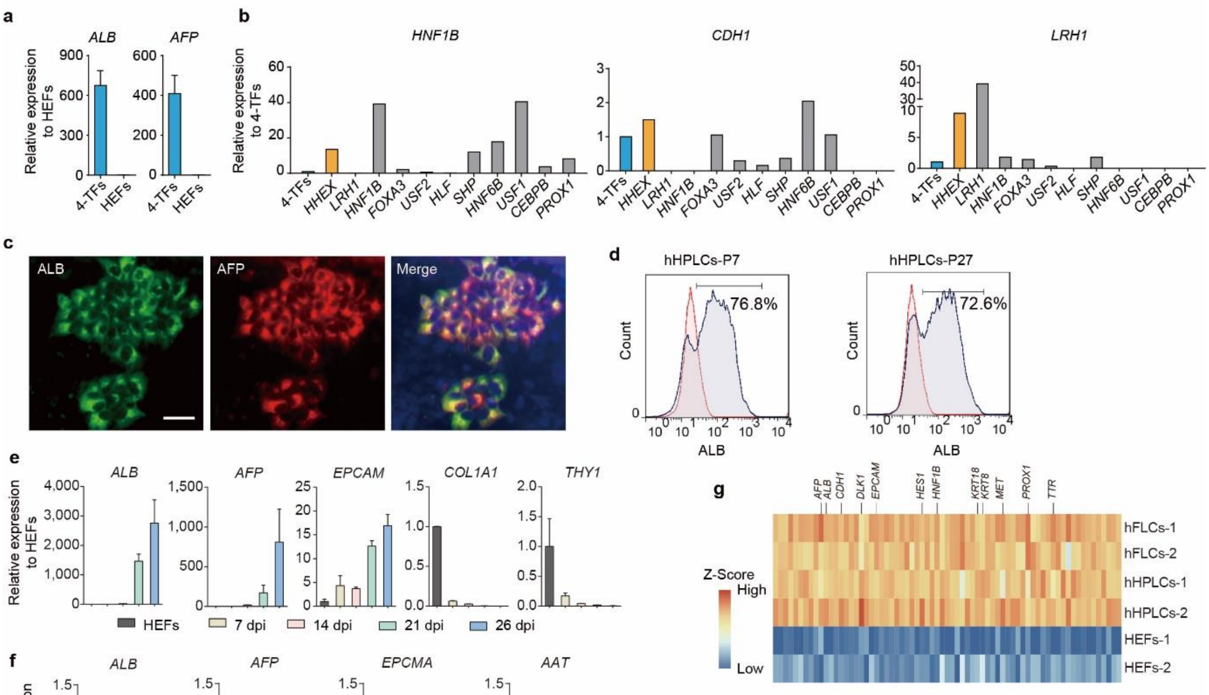

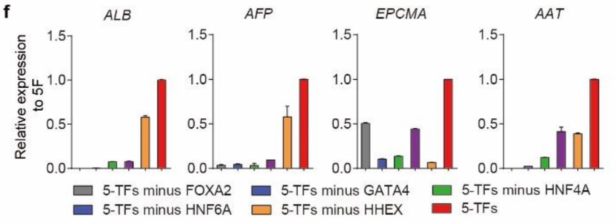

h

Karyotype of hHPLCs

hHPLCs	Passage	Normal cells/total cells	Ratios
hHPLCs-1	P4	50/50	100%
	P29	50/50	100%
hHPLCs-2	P5	50/50	100%
	P30	50/50	100%
hHPLCs-3	P1	50/50	100%
	P30	47/50	94%

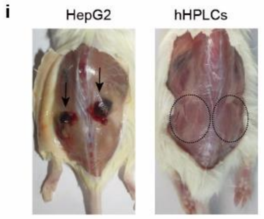

j

Cell type	Tumor formation/ total injection sites
HepG2	6/6
hHPLCs	0/24

Figure S1. Generation of hHPLCs by defined factors *in vitro*. (a) The expression of *ALB* and *AFP* in HEFs and HEFs overexpressed with 4-TFs at 16 dpi. *n* = 3. Relative expression was normalized HEFs. (**b**) Transcription factors screening based on 4-TFs evaluated by RT-qPCR analysis of gene expression. Relative expression was normalized to HEFs overexpressed with 4-TFs at 16 dpi. **(c**) Immunofluorescence staining of ALB- and AFP-positive colonies in 5 TF-overexpressing HEFs at 15 dpi. **(d)** Flow cytometry analysis of ALB positive cells in hHPLCs at P7 and P27. **(e**) Dynamic gene expression analysis of human hepatic progenitor markers *ALB, AFP, EPCAM* as well as fibroblast markers *COL1A1* and *THY1* in reprogrammed cells at different time points by RT-qPCR. Relative expression was normalized to HEFs. *n* = 2. **(f**) Gene expression analysis of human hepatic progenitor markers in reprogramming cells overexpressed with 5-TFs and "5-TFs minus one" at 20 dpi. *n* = 3. **(g)** Sets of human hepatic progenitor-enriched genes were analyzed among hHPLCs, HEFs, and hFLCs. (**h**) Karyotype of three hHPLC cell lines in early and late passages. **(i)** hHPLCs or HepG2 were subcutaneously transplanted to the left and right side of NPG mice. Tumors were indicated by arrows. Transplantation sites were indicated by dashed circles. **(j)** Summary of the tumor formation of NPG mice subcutaneously transplanted with hHPLCs and HepG2 cells. No tumor was found in hHPLCs and hiHeps group over 12 weeks post transplantation. HepG2-derived tumors were found during 2-3 weeks. The scale bars represent 50 μm. Data are presented as mean ± SEM.
