# Supplementary material for: A two-step lineage reprogramming strategy to generate functionally competent human hepatocytes from fibroblasts
Source: Cell Res. 2019 Jul 3;29(9):696–710. doi: 10.1038/s41422-019-0196-x (PMC6796870; doi:10.1038/s41422-019-0196-x)
Supplement: Supplementary file 2 — Supplementary information, Figure S2 [file 41422_2019_196_MOESM2_ESM.pdf]

Figure S2

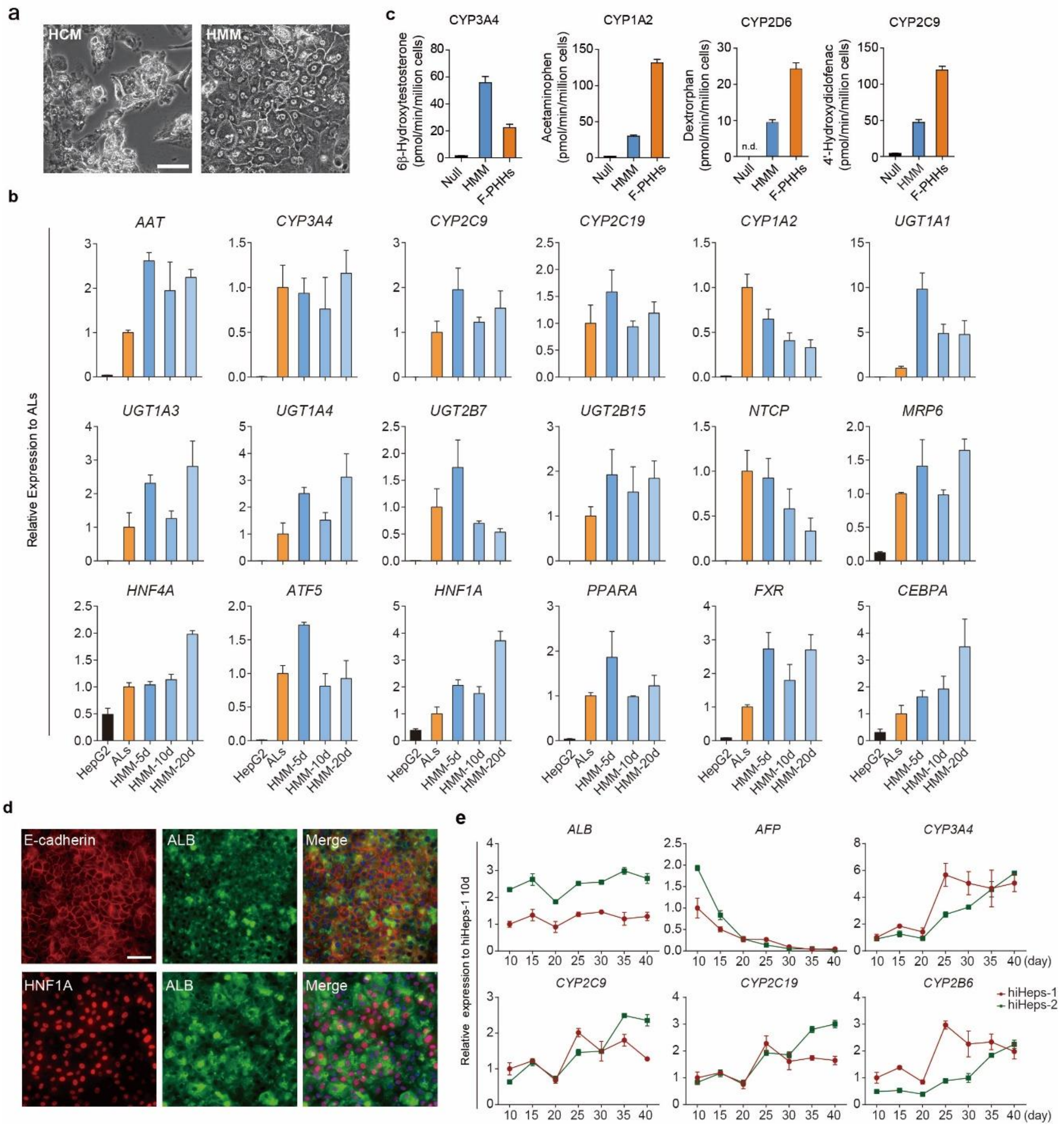
a
HCM
HMM
c
CYP3A4
CYP1A2
CYP2D6
CYP2C9
6β-Hydroxytestosterone (pmol/min/million cells)
Acetaminophen (pmol/min/million cells)
Dextrorphan (pmol/min/million cells)
4'-Hydroxydiclofenac (pmol/min/million cells)
n.d.
Null
HMM
F-PHHs
b
Relative Expression to ALs
AAT
CYP3A4
CYP2C9
CYP2C19
CYP1A2
UGT1A1
UGT1A3
UGT1A4
UGT2B7
UGT2B15
NTCP
MRP6
HNF4A
ATF5
HNF1A
PPARA
FXR
CEBPA
HepG2
ALs
HMM-5d
HMM-10d
HMM-20d
d
E-cadherin
ALB
Merge
HNF1A
e
Relative expression to hiHeps-1 10d
ALB
AFP
CYP3A4
CYP2C9
CYP2C19
CYP2B6
hiHeps-1
hiHeps-2
(day)

Figure S2. Characterization of HMM-maintained primary hepatocytes and hiHeps. (**a**) Morphology of PHHs after two weeks of culturing under different conditions. (**b**) RT-qPCR analyses of key hepatic functional markers and transcription factors in HMM-cultured hepatocytes (n = 3), ALs (n = 4) and HepG2 cells (n = 2). Relative expression was normalized to ALs. (**c**) Drug-metabolizing activity analyses of hepatocytes cultured in HMM for 30 days. UPLC/MS/MS was used to analyze the activities of CYP3A4, CYP1A2 and CYP2D6, CYP2C9. n = 3. (**d**) Co-immunofluorescence staining of E-cadherin (CDH1) and HNF1A with ALB in hiHeps derived from hHPLCs-P26. (**e**) Analysis of the long-term expression of key hepatic genes in hiHeps cultured in HMM by RT-qPCR. Relative expression was normalized to hiHeps-1 cultured in HMM for 10 days. n = 3. The scale bars represent 50 μm. Data are presented as mean ± SEM. Data not detected, "n.d.".
