# Supplementary material for: A two-step lineage reprogramming strategy to generate functionally competent human hepatocytes from fibroblasts
Source: Cell Res. 2019 Jul 3;29(9):696–710. doi: 10.1038/s41422-019-0196-x (PMC6796870; doi:10.1038/s41422-019-0196-x)
Supplement: Supplementary file 3 — Supplementary information, Figure S3 [file 41422_2019_196_MOESM3_ESM.pdf]

Figure S3

a

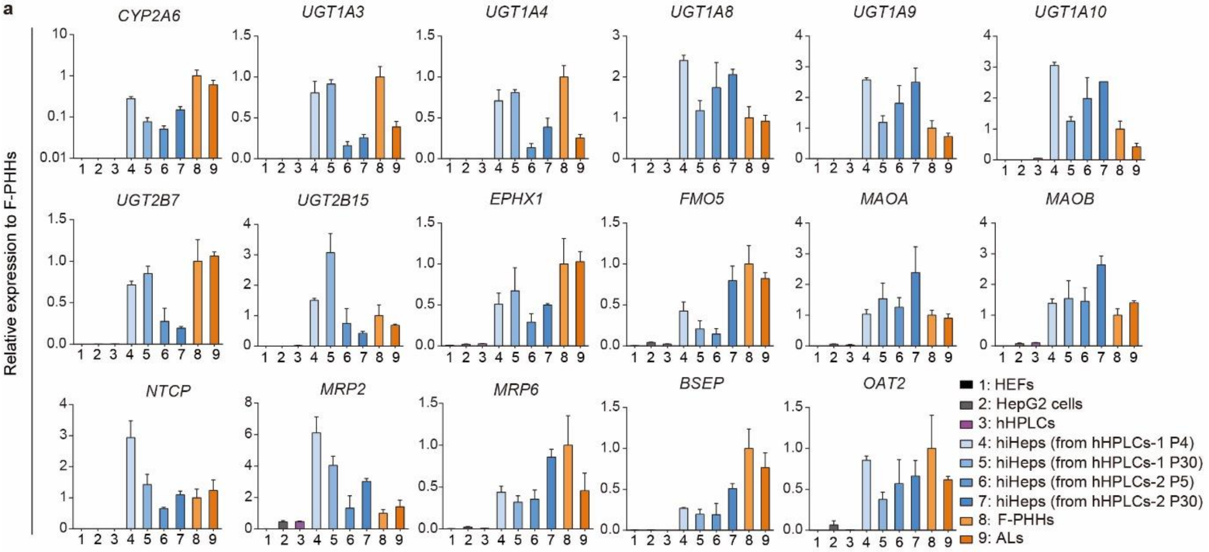

b

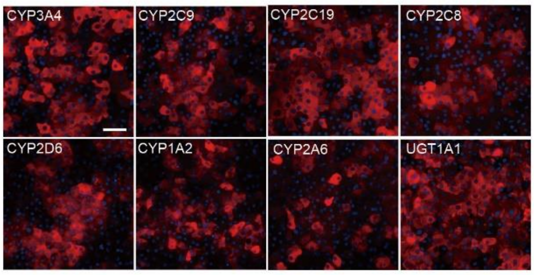

c

Drugs	Responsible Enzymes	*In vitro* CL_{int} by hiHeps (μL/min/10^6)	Scaled *in vivo* CL_h by hiHeps (mL/min/kg)	Obeserved CL_h *in vivo* (mL/min/kg)	Prediction range
Midazolam	CYP3A4	2.8	5.3	5.3	within 2 fold
Verapamil	CYP3A4	7.8	10.1	18	within 2 fold
Diclofenac	CYP2C9	4.5	7.4	7.6	within 2 fold
Phenacetin	CYP1A2	18.3	14.1	21	within 2 fold
Naloxone	UGT2B7	14.2	13	18	within 2 fold

d

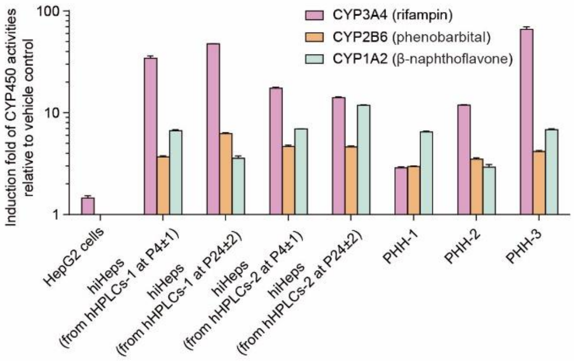

e

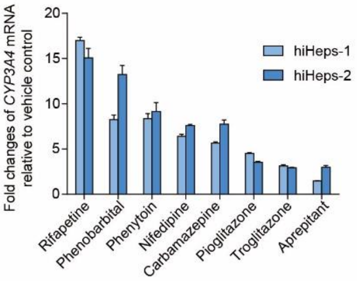

Figure S3. hiHeps show drug-metabolizing ability. (a) RT-qPCR analysis of major mature hepatocyte functional genes, including phase II drug-metabolizing enzymes and transporters, in HEFs (*n* = 3), HepG2 cells (*n* = 2), hHPLCs (*n* = 2), hiHeps derived from hHPLCs at different passages (*n* = 2), F-PHHs (*n* = 5) and ALs (*n* = 4). Relative expression was normalized to F-PHHs. (**b**) Immunofluorescence staining of 7 key CYP450 enzymes (CYP3A4, CYP2C9, CYP2C19, CYP2C8, CYP2D6, CYP1A2 and CYP2A6) and UGT1A1 in hiHeps derived from hHPLCs at P26. (**c**) UPLC/MS/MS analysis of hepatic clearance of 5 typical drugs by hiHeps. The CL_{int} values of hiHeps *in vitro* were determined and scaled into CL_h *in vivo* by known physiological parameters and projection models. The observed CL_h *in vivo* values were collected from previous reports. (**d**) Induction of CYP3A4 (testosterone), CYP1A2 (phenacetin) and CYP2B6 (bupropion) activities in response to rifampin, β-naphthoflavone and phenobarbital in hiHeps, PHHs and HepG2 cells were analyzed by UPLC/MS/MS. hiHeps derived from both early (P4 ± 1) and late (P24 ± 2) passages of hHPLCs were analyzed. *n* = 3. (**e**) Fold changes of *CYP3A4* expression in hiHeps in response to structurally different inducers. Expression was normalized to vehicle-treated controls. The scale bars represent 50 µm. Data are presented as mean ± SEM.
