# Supplementary material for: A two-step lineage reprogramming strategy to generate functionally competent human hepatocytes from fibroblasts
Source: Cell Res. 2019 Jul 3;29(9):696–710. doi: 10.1038/s41422-019-0196-x (PMC6796870; doi:10.1038/s41422-019-0196-x)
Supplement: Supplementary file 4 — Supplementary information, Figure S4 [file 41422_2019_196_MOESM4_ESM.pdf]

Figure S4

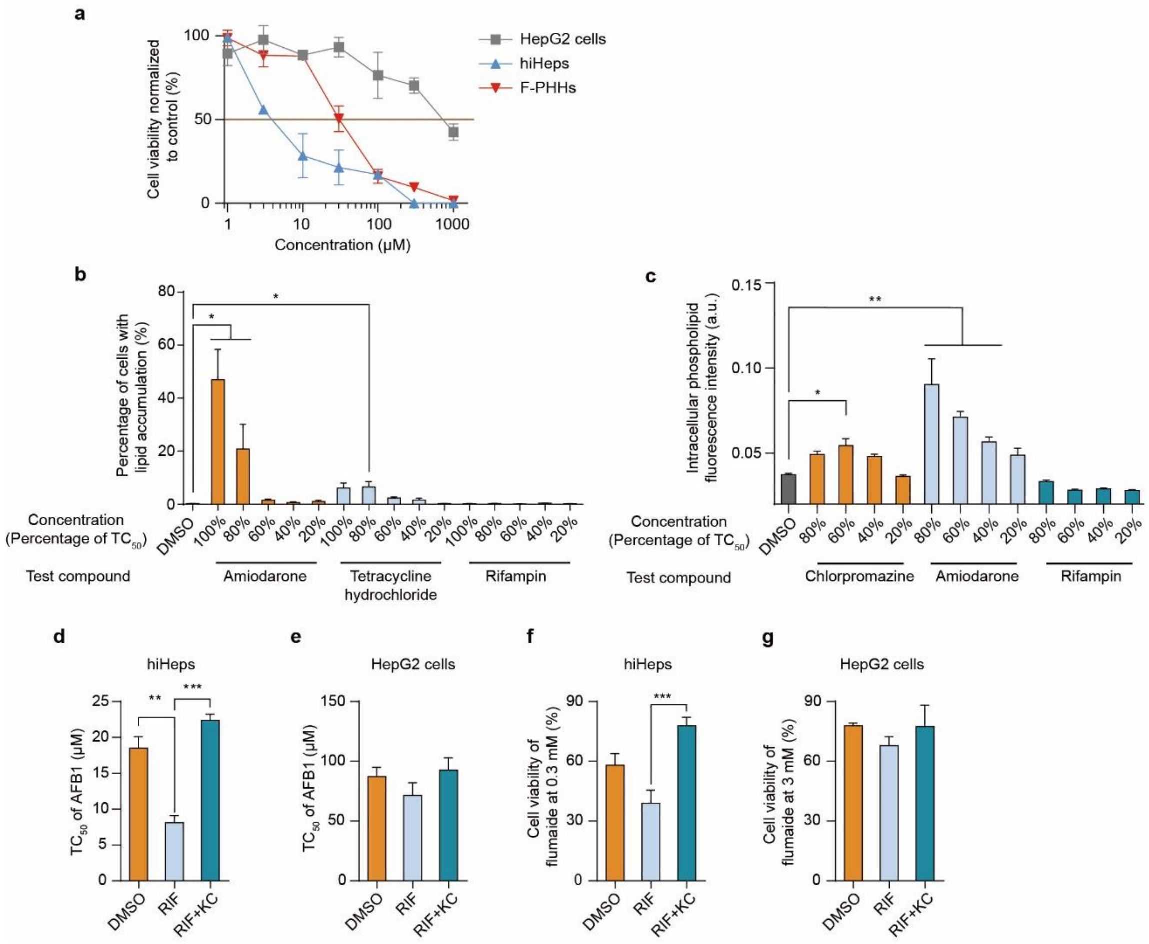
a
Cell viability normalized to control (%)
Concentration (μM)
HepG2 cells
hiHeps
F-PHHs
b
Percentage of cells with lipid accumulation (%)
Concentration (Percentage of TC50)
Test compound
Amiodarone
Tetracycline hydrochloride
Rifampin
c
Intracellular phospholipid fluorescence intensity (a.u.)
Chlorpromazine
Amiodarone
Rifampin
d
hiHeps
TC50 of AFB1 (μM)
e
HepG2 cells
f
hiHeps
Cell viability of flumaide at 0.3 mM (%)
g
HepG2 cells
Cell viability of flumaide at 3 mM (%)
DMSO
RIF
RIF+KC

Figure S4. hiHeps show toxicity prediction ability, Related to Figure 5. (a) Dose-dependent viability curves of hiHeps, F-PHHs and HepG2 cells treated with Aflatoxin B1 (AFB1). The concentration that was calculated to cause a 50% decrease in cell viability (brown line) was determined as the TC_{50}. All data were normalized to cultures treated with vehicle control. **(b-c**) Quantification of dose-dependent steatosis and phospholipidosis in hiHeps after exposure to steatosis/phospholipidosis-causing compounds (Figure 5C), rifampin (non-steatosis/phospholipidosis-causing compound) or DMSO. $n = 4$; a.u., arbitrary units. **(d-g**) Drug-drug interactions mediated toxicity. Toxicity of AFB1 (presented by TC_{50} value) and flutamide (presented by cell viability at 0.3 mM or 3 mM) in hiHeps (d, f), and HepG2 cells (e, g) after treatment with DMSO, the CYP3A4 inducer rifampin (RIF) or the combination of RIF and CYP3A4 inhibitor ketoconazole (KC). $n = 3$ for AFB1 and $n = 6$ for flutamide in both cell types. Data are presented as mean ± SEM. One-way ANOVA was performed. $^*P < 0.05$; $^{**} P < 0.01$; $^{***} P < 0.001$.
