# Supplementary material for: A two-step lineage reprogramming strategy to generate functionally competent human hepatocytes from fibroblasts
Source: Cell Res. 2019 Jul 3;29(9):696–710. doi: 10.1038/s41422-019-0196-x (PMC6796870; doi:10.1038/s41422-019-0196-x)
Supplement: Supplementary file 5 — Supplementary information, Figure S5 [file 41422_2019_196_MOESM5_ESM.pdf]

Figure S5

a

Characterizations of hiHeps	hiHeps derived from early passages (around P5) of hHPLCs	hiHeps derived from late passages (around P25) of hHPLCs
Morphology	Fig. 3a	Fig. 3a
IF staining of hepatocyte markers	Fig. 3c	Supplementary information Fig. S2d
Flow cytometry analysis of ALB positive cells	Fig. 3b	Fig. 3b
RT-qPCR of key hepatic functional genes	Fig. 3d, 4a, Supplementary information Fig. S3a	Fig. 3d, 4a, Supplementary information Fig. S3a
IF staining of key enzymes for drug metabolism	Fig. 4b	Supplementary information Fig. S3b
Global gene expression profile	Fig. 3e	Fig. 3e
ALB secretion	Fig. 3i	Fig. 3i
Metabolic activities of CYP450s	Fig. 4c	Fig. 4c

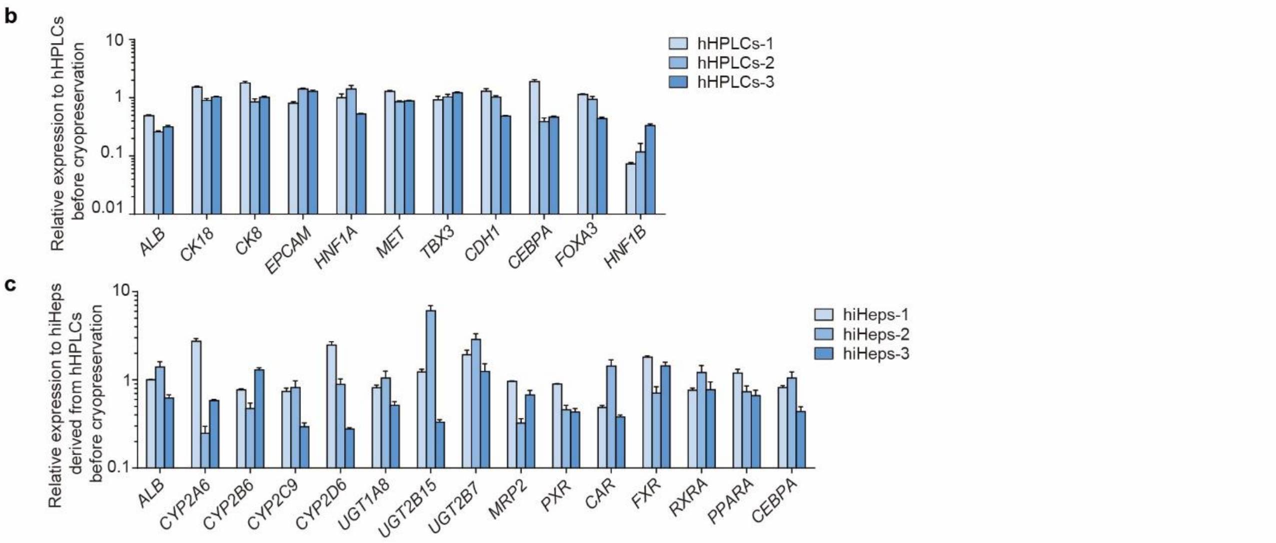

Figure S5. Characteristics of hiHeps derived from early and late passages of hHPLCs and cryopreserved hHPLCs. (a) A summary of the characterization, including the morphology, hepatic functional gene expression and hepatocyte functionality, of hiHeps derived from early and late passages of hHPLCs. **(b**) Gene expression analysis of key human hepatic progenitor markers in hHPLCs before and after cryopreservation by RT-qPCR. *n* = 3. **(c**) Gene expression analysis of hepatocyte functional markers in hiHeps derived from pre-cryopreserved and post-cryopreserved hHPLCs by RT-qPCR. *n* = 3. Data are presented as mean ± SEM.
