# Supplementary material for: A two-step lineage reprogramming strategy to generate functionally competent human hepatocytes from fibroblasts
Source: Cell Res. 2019 Jul 3;29(9):696–710. doi: 10.1038/s41422-019-0196-x (PMC6796870; doi:10.1038/s41422-019-0196-x)
Supplement: Supplementary file 6 — Supplementary information, Figure S6 [file 41422_2019_196_MOESM6_ESM.pdf]

Figure S6

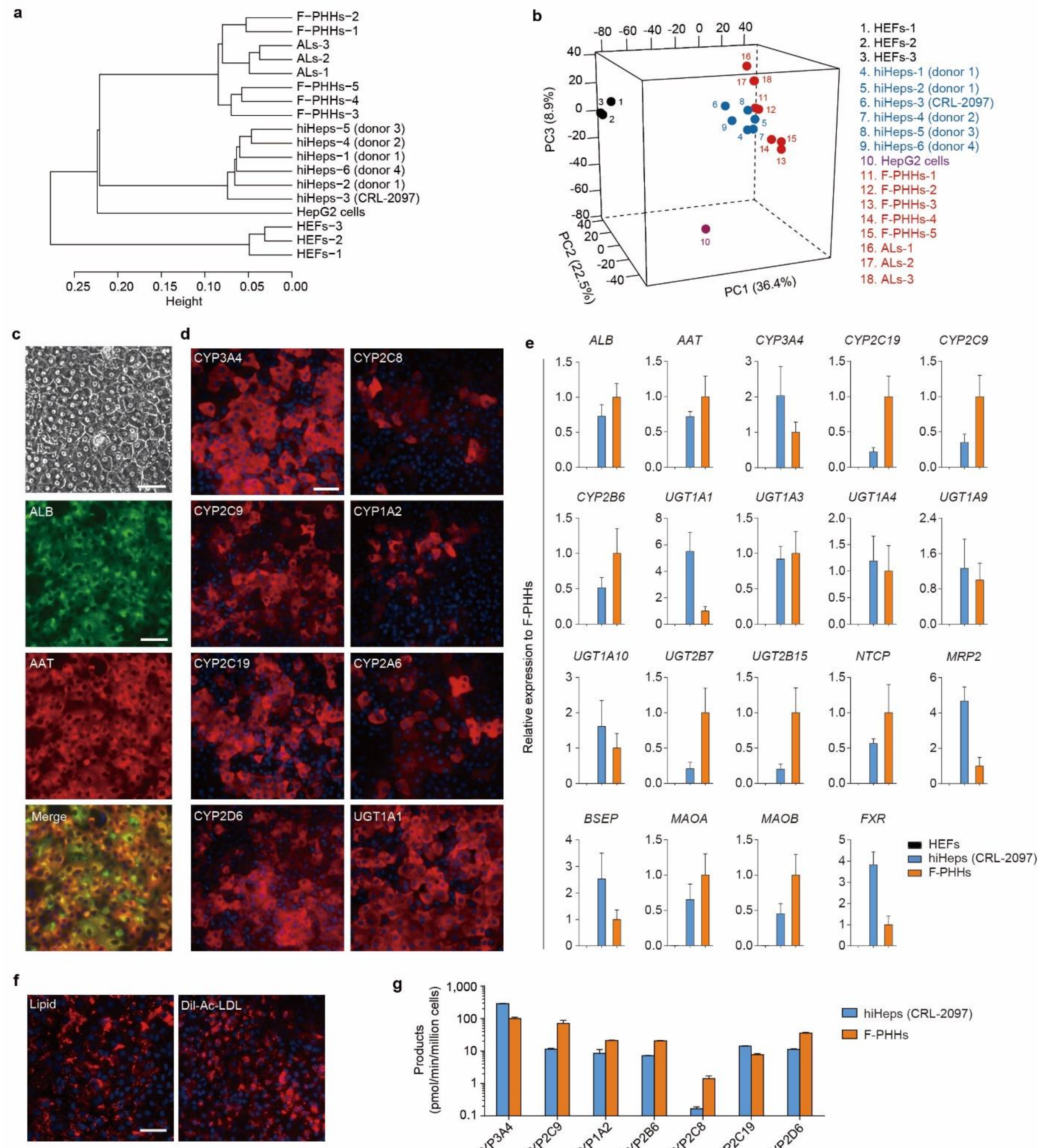

a
F−PHHs−2
F−PHHs−1
ALs−3
ALs−2
ALs−1
F−PHHs−5
F−PHHs−4
F−PHHs−3
hiHeps−5 (donor 3)
hiHeps−4 (donor 2)
hiHeps−1 (donor 1)
hiHeps−6 (donor 4)
hiHeps−2 (donor 1)
hiHeps−3 (CRL-2097)
HepG2 cells
HEFs−3
HEFs−2
HEFs−1
Height
b
PC1 (36.4%)
PC2 (22.5%)
PC3 (8.9%)
1. HEFs-1
2. HEFs-2
3. HEFs-3
4. hiHeps-1 (donor 1)
5. hiHeps-2 (donor 1)
6. hiHeps-3 (CRL-2097)
7. hiHeps-4 (donor 2)
8. hiHeps-5 (donor 3)
9. hiHeps-6 (donor 4)
10. HepG2 cells
11. F-PHHs-1
12. F-PHHs-2
13. F-PHHs-3
14. F-PHHs-4
15. F-PHHs-5
16. ALs-1
17. ALs-2
18. ALs-3
c
ALB
AAT
Merge
d
CYP3A4
CYP2C8
CYP2C9
CYP1A2
CYP2C19
CYP2A6
CYP2D6
UGT1A1
e
Relative expression to F-PHHs
ALB
AAT
CYP3A4
CYP2C19
CYP2C9
CYP2B6
UGT1A1
UGT1A3
UGT1A4
UGT1A9
UGT1A10
UGT2B7
UGT2B15
NTCP
MRP2
BSEP
MAOA
MAOB
FXR
HEFs
hiHeps (CRL-2097)
F-PHHs
f
Lipid
Dil-Ac-LDL
g
Products (pmol/min/million cells)
hiHeps (CRL-2097)
F-PHHs
CYP3A4
CYP2C9
CYP1A2
CYP2B6
CYP2C8
CYP2C19
CYP2D6

Figure S6. Generation of functionally competent hiHeps from additional donors. **(a)** Hierarchical clustering of global gene expression of HEFs, HepG2 cells, hiHeps derived from fibroblasts of different donors, F-PHHs and ALs. **(b)** Principle component analysis of global gene expression of HEFs, HepG2 cells, hiHeps derived from fibroblasts of different donors, F-PHHs and ALs. **(c)** Bright field image of the polygonal morphology of hiHeps derived from CRL-2097 (upper). Co-immunofluorescence staining of ALB and AAT in hiHeps derived from CRL-2097. **(d)** Immunofluorescence staining of 7 key CYP450 enzymes (CYP3A4, CYP2C9, CYP2C19, CYP2C8, CYP2D6, CYP1A2 and CYP2A6) and UGT1A1 in hiHeps derived from CRL-2097. **(e)** RT-qPCR analysis of major mature hepatocyte functional genes in HEFs (n = 3), hiHeps (n = 3) derived from CRL-2097 and F-PHHs (n = 5). Relative expression was normalized to F-PHHs. **(f)** Lipid synthesis and LDL uptake in hiHeps derived from CRL-2097. **(g)** UPLC/MS/MS analysis of drug-metabolic activities of 7 CYP450s in hiHeps derived from CRL-2097 and F-PHHs. Results are presented as pmol/min per million cells. n = 3. The scale bars represent 50 μm. Data are presented as mean ± SEM.
