# Supplementary material for: A two-step lineage reprogramming strategy to generate functionally competent human hepatocytes from fibroblasts
Source: Cell Res. 2019 Jul 3;29(9):696–710. doi: 10.1038/s41422-019-0196-x (PMC6796870; doi:10.1038/s41422-019-0196-x)
Supplement: Supplementary file 7 — Supplementary information, Table S1 [file 41422_2019_196_MOESM7_ESM.pdf]

Table S1. Candidate TFs for screening.

Number	Gene symbol	Accession
1	*FOXA2*	NM_021784
2	*HHEX*	NM_002729
3	*GATA4*	NM_002052
4	*ONECUT1*	NM_004498
5	*HNF4A*	NM_178849
6	*HNF1B*	NM_000458
7	*NR5A2*	NM_205860
8	*PROX1*	NM_001270616
9	*ONECUT2*	NM_004852
10	*FOXA3*	NM_004497
11	*USF2*	NM_003367
12	*HLF*	NM_002126
13	*USF1*	NM_007122
14	*SHP*	NM_021969
15	*CEBPB*	NM_005194
