# Supplementary material for: A two-step lineage reprogramming strategy to generate functionally competent human hepatocytes from fibroblasts
Source: Cell Res. 2019 Jul 3;29(9):696–710. doi: 10.1038/s41422-019-0196-x (PMC6796870; doi:10.1038/s41422-019-0196-x)
Supplement: Supplementary file 8 — Supplementary information, Table S2 [file 41422_2019_196_MOESM8_ESM.pdf]

Table S2. Media tested for hepatic progenitor maintenance or expansion.

Num.	Basal medium	Key components	Reference
1	WEM	ITS, BSA, Nicotinamide, $NaHCO_3$, 2-phospho-l-ascorbic acid, Glucose, GlutaMAX, EGF, Dexamethasone	L ázaro *et al*. 2003[1]
2	DMEM/F12	ITS, EGF, BSA, β-Mercaptoethanol, GlutaMAX, Dexamethasone, Nicotinamide	Kubota and Reid. 2000[2]
3	DMEM/F12	N2, B27, EGF, HGF, bFGF, CHIR99021, A-83-01, Nicotinamide, Forskolin	Huch, Gehart *et al*. 201[3]
4	DMEM/F12	FBS, ITS, EGF, HGF, β-Mercaptoethanol, Dexamethasone, Nicotinamide,	Yu, He *et al*. 2013[4]
5	DMEM/F12	BSA, ITS, EGF, Dexamethasone, Nicotinamide, 2-phospho-l-ascorbic acid	Chen, Kon *et al*. 2007[5]
6	DMEM/F12	FBS, Insulin, HEPES, EGF, HGF, Dexamethasone, Nicotinamide	Rountree, Barsky *et al*. 2007[6]
7	DMEM	FBS	
8	WEM	FBS, HEPES, Nicotinamide, 2-phospho-l-ascorbic acid, $NaHCO_3$, Glucose, ITS, EGF, HGF, Dexamethasone	Okabe, Tsukahara *et al*. 2009[7]
9	DMEM	FBS, EGF, bFGF	Oertel, Menthena *et al*. 2008[8]
10	DMEM/F12	ITS, EGF, E-616452, CHIR99021, Lysophosphatidic acid, Sphingosine 1-phosphate, Nicotinamide, 2-phospho-l-ascorbic acid	Lv, Han *et al*. 2015[9]

Reference

1 Lazaro, C. A. *et al.* Establishment, characterization, and long-term maintenance of cultures of human fetal hepatocytes. *Hepatology* **38**, 1095-1106, doi:10.1053/jhep.2003.50448 (2003).

2 Kubota, H. & Reid, L. M. Clonogenic hepatoblasts, common precursors for hepatocytic and biliary lineages, are lacking classical major histocompatibility complex class I antigen. *Proceedings of the National Academy of Sciences of the United States of America* **97**, 12132-12137, doi:10.1073/pnas.97.22.12132 (2000).

3 Huch, M. *et al.* Long-term culture of genome-stable bipotent stem cells from adult human liver. *Cell* **160**, 299-312, doi:10.1016/j.cell.2014.11.050 (2015).

4 Yu, B. *et al.* Reprogramming fibroblasts into bipotential hepatic stem cells by defined factors. *Cell stem cell* **13**, 328-340, doi:10.1016/j.stem.2013.06.017 (2013).

5 Chen, Q., Kon, J., Ooe, H., Sasaki, K. & Mitaka, T. Selective proliferation of rat hepatocyte progenitor cells in serum-free culture. *Nature protocols* **2**, 1197-1205, doi:10.1038/nprot.2007.118 (2007).

6 Rountree, C. B. *et al.* A CD133-expressing murine liver oval cell population with bilineage potential. *Stem cells* **25**, 2419-2429, doi:10.1634/stemcells.2007-0176 (2007).

7 Okabe, M. *et al.* Potential hepatic stem cells reside in EpCAM+ cells of normal and injured mouse liver. *Development* **136**, 1951-1960, doi:10.1242/dev.031369 (2009).

8 Oertel, M. *et al.* Purification of fetal liver stem/progenitor cells containing all the repopulation potential for normal adult rat liver. *Gastroenterology* **134**, 823-832, doi:10.1053/j.gastro.2008.01.007 (2008).

9 Lv, L. *et al.* Self-renewal of hepatoblasts under chemically defined conditions by iterative growth factor and chemical screening. *Hepatology* **61**, 337-347, doi:10.1002/hep.27421 (2015).
