# Supplementary material for: A two-step lineage reprogramming strategy to generate functionally competent human hepatocytes from fibroblasts
Source: Cell Res. 2019 Jul 3;29(9):696–710. doi: 10.1038/s41422-019-0196-x (PMC6796870; doi:10.1038/s41422-019-0196-x)
Supplement: Supplementary file 9 — Supplementary information, Table S3 [file 41422_2019_196_MOESM9_ESM.pdf]

Table S3. Donor information of the human fibroblasts.

Batch ID	Age	Gender	Race	Tissue
6M321A11	11w	Male	Asian	Skin
CRL2097	Newborn	Male	Unknown	Skin
5M427A12	12.5w	Male	Asian	Skin
5M625A15	15w	Male	Asian	Skin
6M322A13	13w	Male	Asian	Skin
6M317A17	17w	Male	Asian	Skin
M827AU	Unknown	Female	Asian	Skin
