# Supplementary material for: A two-step lineage reprogramming strategy to generate functionally competent human hepatocytes from fibroblasts
Source: Cell Res. 2019 Jul 3;29(9):696–710. doi: 10.1038/s41422-019-0196-x (PMC6796870; doi:10.1038/s41422-019-0196-x)
Supplement: Supplementary file 10 — Supplementary information, Table S4 [file 41422_2019_196_MOESM10_ESM.pdf]

Table S4. Donor information of the human primary hepatocytes and liver tissues.

Batch ID	Age	Gender	Race	HBV	Reason for liver resection	Disease state
1DC (Commercial)	74	Male	Caucasian	-	Unknown	Unknown
10DC (Commercial)	Mix	Mix	Mix	-	Unknown	Unknown
6CF420A50	50	Female	Asian	-	Hilar cholangiocarcinoma	A
6CF612A28	28	Female	Asian	-	Hepatoma	A
6CM1010A50	50	Male	Asian	-	Hepatolithiasis	A
6CM1013A56	56	Male	Asian	-	Hepatoma	A
7CM224A35	35	Male	Asian	-	Hepatic hemangioma	A
7CM55A64	64	Male	Asian	-	Cholangiocarcinoma	A
7CF93A38	38	Female	Asian	-	Hepatic hemangioma	A
7CM912A56	56	Male	Asian	-	Hepatoma	A
7CM1222A60	60	Male	Asian	-	Hepatoma	B
8CM619A47	47	Male	Asian	+	Hepatoma	A
8CF710A55	55	Female	Asian	-	Hepatic echinococcosis	A
8CF724A31	31	Female	Asian	-	Hepatic cystadenocarcinoma	A
8TF54A62	62	Female	Asian	-	Hepatic cystadenocarcinoma	A
8TM619A47	47	Male	Asian	+	Hepatocellular carcinoma	A
8TF710A55	55	Female	Asian	-	Hepatic echinococcosis	A
8TM87A48	48	Male	Asian	-	Intrahepatic cholangiocarcinoma	A
