# Supplementary material for: A two-step lineage reprogramming strategy to generate functionally competent human hepatocytes from fibroblasts
Source: Cell Res. 2019 Jul 3;29(9):696–710. doi: 10.1038/s41422-019-0196-x (PMC6796870; doi:10.1038/s41422-019-0196-x)
Supplement: Supplementary file 11 — Supplementary information, Table S5 [file 41422_2019_196_MOESM11_ESM.pdf]

Table S5. Primers used for RT-qPCR.

Gene	Forward Primer (5' to 3')	Reverse Primer (5' to 3')
RPL13A	AGATGGCGGAGGTGCAG	GGCCCAGCAGTACCTGTTTA
RRN18S	GTAACCCGTTGAACCCCATT	CCATCCAATCGGTAGTAGCG
COL1A1	CACACGTCTCGGTCATGGTA	AAGAGGAAGGCCAAGTCGAG
EPCAM	TGATCCTGACTGCGATGAGAG	CTTGTCTGTTCTTCTGACCCC
THY1	GGGAGACCTGCAAGACTGTT	CGGAAGACCCCAGTCCA
CK8	GATTGAGGGCCTCAAAGGC	ACAACTTGGCGTTGGCATC
CK18	TGGTCACCACACAGTCTGCTG	TCTCATGGAGTCCAGGTCGAT
HNF1B	GCACCTCTCCCAGCATCTCA	GTCGGAGGATCTCTCGTTGC
DLK1	GGGCACAGGAGCATTCATAG	GACGGGGAGCTCTGTGATAG
CDH1	GCCGAGAGCTACACGTTCAC	GTCGAGGGAAAAATAGGCTG
MET	GCTGCAAAGCTGTGGTAAACT	CTCCAGCATTTTTACGGACC
LRH1	CGAGTGGGCCAGGAGTAGTA	CGGTAAATGTGGTCGAGGAT
ALB	GCACAGAATCCTTGGTGAACAG	ATGGAAGGTGAATGTTTCAGCA
AFP	CCCGAACTTTCCAAGCCATA	TACATGGGCCACATCCAGG
AAT	ACGAGACAGAAGACGGCATT	CCACTGCTTAAATACGGACGA
HNF1A	CCATCCTCAAAGAGCTGGAG	GTGCTGCTGCAGGTAGGACT
CEBPA	ACAAGAACAGCAACGAGTACCG	CATTGTCACTGGTCAGCTCCA
FOXA3	GAGATGCCGAAGGGGTATCG	TGATTCTCCCGGTAGTAAGGG
TBX3	GCCATGTACGTGTAGGGGTA	CTTCCACCTCCAGCAGCA
ATF5	CTATGAGGTCCTTGGGGGAG	CTCGCTCAGTCATCCAGTCA
CYP1A2	CTTCGTAAACCAGTGGCAGG	AGGGCTTGTTAATGGCAGTG
CYP2A6	GAGTTCCTGTCACTGTTGCG	GTCCTGGCAGGTGTTTCATC
CYP2B6	CCGGGGATATGGTGTGATCTT	CCGAAGTCCCTCATAGTGGTC
CYP2C19	GAAGAGGAGCATTGAGGACCG	GCCCAGGATGAAAGTGGGAT
CYP2C8	CTCGGGACTTTATGGATTGC	CAGTGCCAACCAAGTTTTCA
CYP2C9	GCCACATGCCCTACACAGATG	TAATGTCACAGGTCACTGCATGG
CYP2D6	GTGTCCAACAGGAGATCGACG	CACCTCATGAATCACGGCAGT
CYP3A4	AGCCTGGTGCTCCTCTATCT	CCCTTATGGTAGGACAAAAT
OAT2	CACACTCCATCCAGCAAGG	TTGTACCCTACGGTGCTCAG
MRP2	GGGATCTCTTCCACACTGGAT	CATACAGGCCCTGAAGAGGA
MRP6	AAGAACTTGTTTCCCGGCTT	CTCGGTCTCTGGAGCCTTC
NTCP	AGGGGGACATGAACCTCAG	AGGTCCCCATCATAGATCCC
BSEP	CCAGGAAAAGCATGTGTGAA	TGATCATTTCGCTCTCGATG
FMO5	AGGCCAACTCTTGCAATCAT	TTCACTCTGTGAGGGCAATG
MAOA	TCAGCCAAAGCATGGAGAAT	CAGTCAAGAGTTTGGCAGCA
MAOB	CAACAAATGCGACGTGGTC	GTAAGTCCTGCCTCCCACAC
EPHX1	GTCATCTCCTACTGGCGGAA	CTTCACGTGGATGAAGTGGA
UGT1A1	CCATCATGCCCAATATGGTT	CCACAATTCCATGTTCTCCA
UGT1A8	CTGAGACCATTGATCCCAAAG	GGTATCAACTGCCATCAGGG
UGT1A9	ACTATCCCAAACCCGTGATG	ACCACAATTCCATGTTCTCCA

UGT1A3	GCCAACAGGAAGCCACTATC	CAGCAATTGCCATAGCTTTC
UGT1A4	AACGGGAAGCCACTATCTCA	TCAGCAATTGCCATAGCTTTC
UGT1A10	CCACAATTCCATGTTCTCCA	TGATGCCCAACATGATCTTC
UGT2B15	GTTTTCTCTGGGGTCGATGA	ATTTGGCTTCTTGCCATCAA
UGT2B7	AACGTAATTGCATCAGCCCT	GGTCATTCTGGGGTATCCAC
DDX60	AGTCCAGGATAACAGGATGAATGA	GCTCACGCAAGGAAACACTG
IRF7	AGGGTGACAGGTACGGCTCT	CTCCTGGAGAGGGACAAGAA
IRF9	GCCCTACAAGGTGTATCAGTTG	TGCTGTCGCTTTGATGGTACT
PKR	ACTTGGCCAAATCCACCTG	CCCAGATTTGACCTTCCTGA
ZAP	TGTTCAGTCCAGAGAGTTCGTG	GGTGCAACTATTCGCAGTCC
BST2	CTTTTGTCCTTGGGCCTTCT	AGAAGGGCTTTCAGGATGTG
ISG15	TGGACAAATGCGACGAACCTC	TCAGCCGTACCTCGTAGGTG
ISG20	GCTTGCCTTTCAGGAGCTG	ATCACCGATTACAGAACCCG
MX1	GATGATCAAAGGGATGTGGC	AGCTCGGCAACAGACTCTTC
RSAD2	TGGCTCTCCACCTGAAAAGT	GCCAAAACATCCTTTGTGCT
IFIT1	GCCCTATCTGGTGATGCAGT	GCAGCCAAGTTTTACCGAAG
IFITM1	GCCAACCATCTTCCTGTCC	ATGTCGTCTGGTCCCTGTTC
IFITM2	CCAACCATCTTCCTGTCCC	ATGTCGTCTGGTCCCTGTTC
IFITM3	CCAACCATCTTCCTGTCCC	ATGTCGTCTGGTCCCTGTTC
PPARA	AGAGATTTCGCAATCCATCGG	ACTGGTATTCCGTAAAGCCAAAG
CAR	TTGCAGAAGTGCTTAGATGCTG	GCCGACAGTATCATGTCTTTCCT
RXRA	GACGGAGCTTGTGTCCAAGAT	AGTCAGGGTTAAAGAGGACGAT
FXR	CCTGTGAGGGGTGTAAAGGTT	CACTCTTGACACTTTCTTCGCAT
PXR	AAGCCCAGTGTCAACGCAG	AGATTTGGGGACCTCCGACTT
LXR	CCTTCAGAACCCACAGAGATCC	ACGCTGCATAGCTCGTTCC
HNF4A	CGTGGTGGACAAAGACAAGA	CATAGCTTGACCTTCGAGTGC
