# Supplementary material for: A two-step lineage reprogramming strategy to generate functionally competent human hepatocytes from fibroblasts
Source: Cell Res. 2019 Jul 3;29(9):696–710. doi: 10.1038/s41422-019-0196-x (PMC6796870; doi:10.1038/s41422-019-0196-x)
Supplement: Supplementary file 12 — Supplementary information, Table S6 [file 41422_2019_196_MOESM12_ESM.pdf]

Table S6. Antibodies used for immunofluorescence staining.

Protein	Antibody	Catalog Number
ALB	Human Albumin Antibody	A80-129A (Bethyl Laboratories, Inc.)
AAT	AAT antibody	ZA-0007 (ZSGB-BIO)
AFP	Alpha-1-Fetoprotein	ZM-0009 (ZSGB-BIO)
CYP3A4	CYTOCHROME P450 3A4	AHP622Z (BIO-RAD)
CYP1A2	CYTOCHROME P450 1A2	AHP69Z (BIO-RAD)
CYP2C9	CYTOCHROME P450 2C9	AHP617Z (BIO-RAD)
CYP2C19	CYTOCHROME P450 2C19	AHP618Z (AbD Serotec)
CYP2A6	CYTOCHROME P450 2A6	AHP612Z (AbD Serotec)
CYP2E1	CYTOCHROME P450 2E1	AHP621Z (AbD Serotec)
CK18	Mouse anti Human CK18	ZM-0073 (ZSGB-BIO)
CK8	Mouse anti Human CK8	ZM-0310 (ZSGB-BIO)
UGT1A1	Human UGT1A1 Affinity Purified Polyclonal Ab	AF6490 (R&D)
CYP2D6	Rabbit anti Human CYP2D6	HPA045223 (Sigma)
CYP2C8	Rabbit anti Human CYP2C8	AHP614Z (AbD Serotec)
NTCP	Anti-SLC10A1 antibody	HPA042727 (Sigma)
MRP2	Anti-MRP2 antibody	ab3373 (Abcam)
E-Cadherin	E-Cadherin (24E10) Rabbit mAb	#3195 (Cell Signaling Technology)
HBcAg	HBcAg Rabbit Polyclonal Antibody	Z2085(ZETA Corporation)
HNF1A	HNF1A (H-140)	SC-10791 (santa cruz)
CEBPA	CEBPA Rabbit antibody	2295s (Cell signaling Technology)
