# Supplementary material for: A two-step lineage reprogramming strategy to generate functionally competent human hepatocytes from fibroblasts
Source: Cell Res. 2019 Jul 3;29(9):696–710. doi: 10.1038/s41422-019-0196-x (PMC6796870; doi:10.1038/s41422-019-0196-x)
Supplement: Supplementary file 13 — Supplementary information, Table S7 [file 41422_2019_196_MOESM13_ESM.pdf]

Table S7. Drugs used for measurements of CYP450 activity.

CYP450s isoforms	Fraction of metabolizing marketed drugs[1]	Fraction of metabolizing marketed drugs[2]	Standard substrate	Concentration of substrate	Internal standard	Product for detection	FDA concern[3]
CYP3A4	30.2%	50.0%	Testosterone	200 μM	6β-Hydroxytestosterone-[D7]	6β-Hydroxytestosterone	concern
CYP2D6	20.0%	30.0%	Dextromethorphan	15 μM	Dextrorphan-[D3]	Dextrorphan	concern
CYP2C9	12.8%	10.0%	Difclofenac	25 μM	4′-Hydroxydiclofenac-[13C6]	4'-Hydroxydiclofenac	concern
CYP2B6	7.2%	N.A.	Bupropion	500 μM	Hydroxybupropion-[D6]	Hydroxybupropion	concern
CYP1A2	8.9%	4.0%	Phenacetin	100 μM	Acetomidophenol-[13C2, 15N]	Acetaminophen	concern
CYP2C19	6.8%	2.0%	S-mephenytoin	250 μM	4′-Hydroxymephenytoin-[D3]	4'-Hydroxymephenytoin	concern
CYP2C8	4.7%	N.A.	Paclitaxel	20 μM	6α-hydroxypaclitaxel-[D5]	6α-hydroxypaclitaxel	concern

Data not available were labeled as “N.A.”.

Reference

1 Zanger, U. M. & Schwab, M. Cytochrome P450 enzymes in drug metabolism: regulation of gene expression, enzyme activities, and impact of genetic variation. *Pharmacology & therapeutics* **138**, 103-141, doi:10.1016/j.pharmthera.2012.12.007 (2013).

2 Zhou, S. F., Liu, J. P. & Chowbay, B. Polymorphism of human cytochrome P450 enzymes and its clinical impact. *Drug metabolism reviews* **41**, 89-295, doi:10.1080/03602530902843483 (2009).

3 FDA. *Drug Development and Drug Interactions: Table of Substrates, Inhibitors and Inducers*, <https://www.fda.gov/drugs/developmentapprovalprocess/developmentresources/druginteractionslabeling/ucm093664.htm> (2017).
