# Supplementary material for: A two-step lineage reprogramming strategy to generate functionally competent human hepatocytes from fibroblasts
Source: Cell Res. 2019 Jul 3;29(9):696–710. doi: 10.1038/s41422-019-0196-x (PMC6796870; doi:10.1038/s41422-019-0196-x)
Supplement: Supplementary file 14 — Supplementary information, Table S8 [file 41422_2019_196_MOESM14_ESM.pdf]

Table S8. Drugs used for measurements of hepatic clearance.

Drugs	Responsible enzymes for metabolism	Concentration (μM)	Internal standards
Midazolam	CYP3A4	1	Hydroxymidazolam-[13C3]
Verapamil	CYP3A4	1	Verapamil-[D6] hydrochloride
Diclofenac	CYP2C9	1	4′-Hydroxydiclofenac-[13C6]
Phenacetin	CYP1A2	1	Acetomidophenol-[13C2, 15N]
Naloxone	UTG2B7	1	Naloxone-[D5]
