# Supplementary material for: A two-step lineage reprogramming strategy to generate functionally competent human hepatocytes from fibroblasts
Source: Cell Res. 2019 Jul 3;29(9):696–710. doi: 10.1038/s41422-019-0196-x (PMC6796870; doi:10.1038/s41422-019-0196-x)
Supplement: Supplementary file 15 — Supplementary information, Table S9 [file 41422_2019_196_MOESM15_ESM.pdf]

Table S9. Compounds used for toxicity prediction.

Compound	The highest tested concentration (mM)	Label[1,2]	Toxicity mechanism
Acetaminophen	3	Representative drug	Bioactivation; Mitochondrial dysfunction
Aflatoxin B1	1	Representative drug	DNA damage, Bioactivation
Maraviroc	3	DILI concern by FDA	Immunoreaction; Bioactivation
Carbamazepine	3	DILI concern by FDA	Bioactivation; Mitochondrial dysfunction
Bosentan	3	DILI concern by FDA	Direct toxicity; Bioactivation
Etodolac	3	DILI concern by FDA	Idiosyncratic toxicity
Fenoprofen	3	DILI concern by FDA	Idiosyncratic toxicity
Flutamide	3	DILI concern by FDA	Bioactivation
Cyclosporine A	3	DILI concern by FDA	Reactive oxygen species
Trazodone	1.5	DILI concern by FDA	Reactive oxygen species; Bioactivation
Leflunomide	3	DILI concern by FDA	Bioactivation
Rifampin	1.5	DILI concern by FDA	Reactive oxygen species
Tolcapone	3	DILI concern by FDA	Bioactivation; Mitochondrial dysfunction
Diclofenac	3	DILI concern by FDA	Bioactivation; Mitochondrial dysfunction
Tacrine	3	DILI concern by FDA	Reactive oxygen species
Ticlopidine	3	DILI concern by FDA	Idiosyncratic toxicity
Docetaxel	3	DILI concern by FDA	Mitochondrial dysfunction
Zafirlukast	3	DILI concern by FDA	Idiosyncratic toxicity
Simvastatin	3	DILI concern by FDA	Mitochondrial dysfunction
Nortriptyline	3	DILI concern by FDA	Idiosyncratic toxicity
Tipranavir	3	DILI concern by FDA	Bioactivation
Nefazodone	1	DILI concern by FDA	Bioactivation; Mitochondrial dysfunction
Chlorpromazine	3	DILI concern by FDA	Bioactivation; Idiosyncratic toxicity
Amiodarone	0.3	DILI concern by FDA	Phospholipidosis; Steatotic; Mitochondrial dysfunction
Sunitinib	1.5	DILI concern by FDA	Hyperammonemia

Reference

1 Chen, M. *et al.* FDA-approved drug labeling for the study of drug-induced liver injury. *Drug discovery today* **16**, 697-703, doi:10.1016/j.drudis.2011.05.007 (2011).

2 Levy, G. *et al.* Long-term culture and expansion of primary human hepatocytes. *Nat Biotechnol* **33**, 1264-1271, doi:10.1038/nbt.3377 (2015).
